# Post hoc comparison of the intrarenal and circulating renin‐angiotensin(‐aldosterone) systems in cats with ischemia‐induced chronic kidney disease

**DOI:** 10.14814/phy2.70417

**Published:** 2025-06-25

**Authors:** Jane H. C. Huang, Bianca N. Lourenço, Chad W. Schmiedt, Jaime L. Tarigo, Amanda E. Coleman

**Affiliations:** ^1^ Department of Small Animal Medicine and Surgery College of Veterinary Medicine, University of Georgia Athens Georgia USA; ^2^ Department of Pathology College of Veterinary Medicine, University of Georgia Athens Georgia USA

**Keywords:** cat, chronic kidney disease models, kidney, liquid chromatography–tandem mass spectrometry, mRNA, renal ischemia

## Abstract

Activities of the circulating and intrarenal renin‐angiotensin(‐aldosterone) systems (RA[A]S) are incompletely understood in people and cats with chronic kidney disease (CKD). We measured circulating and intrarenal RA(A)S markers in healthy cats (*n* = 8) and cats with induced CKD (*n* = 6 subjected to unilateral renal ischemia [RI group] and *n* = 5 subjected to RI and delayed contralateral nephrectomy [RI‐DCN group]). Serum equilibrium concentrations of angiotensin peptides and aldosterone, plasma renin activity, and urinary aldosterone‐to‐creatinine ratio were evaluated before and after renal injury in CKD cats and at a single timepoint in healthy cats. Renal tissular concentrations of angiotensin peptides and mRNA levels of RA(A)S‐related genes were measured in all cats. There was no significant correlation between circulating angiotensin peptide concentrations and their respective renal concentrations. Intrarenal angiotensin I concentrations and *AGT* transcript levels were positively correlated, and *ACE* transcript levels were negatively correlated with serum creatinine concentration. Circulating RA(A)S markers were not different between healthy and CKD groups, except for serum angiotensin 1–5, which was lower in the RI group compared to the healthy group. Intrarenal angiotensin peptide concentrations did not differ among groups. Compared to healthy cats, mRNA levels of *ACE*, *AT1R*, and *REN* were lower, and *AGT* levels were higher in one or both CKD group(s).

## Graphical abstract

Samples from cats with CKD or healthy were used to compare circulating and intrarenal RA(A)S markers. There was no correlation between circulating concentrations of angiotensin peptides and their respective renal tissular concentrations.

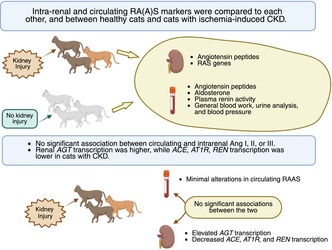

## 1 INTRODUCTION

Excessive activation of the renin‐angiotensin‐aldosterone system (RAAS) plays a critical role in the progression of chronic kidney disease (CKD) (Brewster & Perazella, 2004; Saldanha da Silva et al., 2017) by triggering hemodynamic maladaptation and enhancing inflammation, fibrosis, and oxidative stress within the kidneys (Ba Aqeel et al., 2017; Brewster & Perazella, 2004; Cao et al., 2016; Chen et al., 2016; Chou et al., 2018; Saldanha da Silva et al., 2017). The central role of RAAS in CKD is supported by the clinical benefits of pharmacologic RAAS inhibition in human patients suffering from proteinuric or hypertensive CKD or diabetic nephropathy (Loutradis et al., 2021; Loutradis & Sarafidis, 2020).

CKD is highly prevalent in senior domestic cats (*Felis catus*), affecting 30%–80% of those greater than 10 years of age (Marino et al., 2014; Sparkes et al., 2016). The typical renal histological findings in cats with CKD (i.e., tubulointerstitial inflammation and fibrosis (Brown et al., 2016)) mirror those observed in people with some forms of CKD (Devuyst et al., 2019; Gifford et al., 2017; Joyce et al., 2017; Weaver et al., 2015; Wijkström et al., 2017) and people with advancing CKD stages regardless of etiology (AlQudah et al., 2020; Sethi et al., 2017). Additionally, systemic arterial hypertension is reported in approximately 65% of cats with CKD–a prevalence comparable to that of people with CKD (Kalaitzidis & Elisaf, 2018; Muntner et al., 2010; Stiles, 1994). Because of these shared characteristics, cats have been proposed as a naturally occurring model for human CKD (Lawson et al., 2018; Lourenço et al., 2024), and a greater understanding of CKD in cats can likely benefit patients of both species.

Because inhibitors of RAAS are effective antiproteinuric (King et al., 2006; Mizutani et al., 2006; Sent et al., 2015) and antihypertensive agents (Coleman et al., 2019; Glaus et al., 2019) in cats with CKD, they have become part of the standard‐of‐care for these conditions of this species (International Renal Interest Society 2023). However, studies evaluating the circulating RAAS in affected cats have found inconsistent evidence of its activation (Huang et al., 2024a; Jensen et al., 1997; Jepson et al., 2014; Huang et al., 2024b; Kai et al., 2022; Mathur et al., 2004; Mishina et al., 1998; Steele et al., 2002; Ward et al., 2022; Watanabe & Mishina, 2007). Over the past few decades, attention has increasingly turned to the role of the intrarenal renin‐angiotensin system (RAS) in kidney diseases (Kobori et al., 2007; Yang & Xu, 2017). Angiotensin II concentrations in renal tubular fluid exceed those in circulation by several orders of magnitude, with evidence suggesting that kidney concentrations are not simply a reflection of the degree of circulating RAAS activation and can instead be regulated independently from it (Braam et al., 1993; Navar et al., 1994; Seikaly et al., 1990; Siragy et al., 1995). Studies in human beings have shown activation of the intrarenal RAS in various forms of kidney diseases (Juretzko et al., 2017; Mills et al., 2012; Urushihara et al., 2015; Wu et al., 2019), and that intrarenal RAS markers correlate to renal tissue inflammation (Dou et al., 2019; Wu et al., 2019) and fibrosis (Ohashi et al., 2017), predict future renal function decline (Cui et al., 2018; Dou et al., 2019; Jang et al., 2012; Yamamoto et al., 2007), and respond to RAAS inhibition (Kobori et al., 2009; Mizushige et al., 2016; Nishiyama et al., 2011; Persson et al., 2013; Urushihara et al., 2015; Yamamoto et al., 2007). Our group recently reported increased gene transcript levels of angiotensinogen in the kidneys of cats with naturally occurring CKD compared to those with healthy kidneys (Lourenço et al., 2022). This is consistent with findings in human CKD patients, for whom intrarenal angiotensinogen protein expression was upregulated in one study (Del Prete et al., 2003). While investigators have extensively evaluated the circulating or intrarenal RA(A)S in people with CKD, studies that compare these two systems within individuals are lacking. Whether, how, and to what extent the circulating RAAS and intrarenal RAS interact remains under debate (Nishiyama & Kobori, 2018; Smith & Layton, 2023; Sun et al., 2020). Understanding the absolute and relative activities of these systems in CKD is necessary to clarify its pathophysiology, to optimize treatment strategies, and to design rational studies.

In addition to CKD, aged cats are prone to other diseases–notably, hyperthyroidism‐that impact kidney function (Covey et al., 2019; Stock et al., 2017; Williams et al., 2010). Studies of experimentally induced CKD allow researchers to avoid common relevant comorbidities and to control potentially confounding husbandry factors. The aim of this study was to evaluate markers of both the circulating and intrarenal renin‐angiotensin(‐aldosterone) systems (RA[A]S) in cats with surgically induced CKD and in healthy cats. We hypothesized that compared to healthy cats, cats with CKD would have an intrarenal RAS profile–including differences in concentrations of angiotensin peptides, mRNA transcript levels of RAS‐related genes, or both–supportive of increased activity of this system. Based on data from other species (Braam et al., 1993; Navar et al., 1994; Seikaly et al., 1990; Siragy et al., 1995), we also hypothesized that there would be no or weak correlation between circulating and intrarenal angiotensin peptide concentrations, suggesting “uncoupling” of these systems.

## 2 MATERIALS AND METHODS

### 2.1 Animals

This was a retrospective study which used banked serum, plasma, urine, and renal tissue samples collected from domestic cats during previous studies (Brown et al., 2019; Lourenço et al., 2020). In the original studies, purpose‐bred cats were subjected to either transient unilateral renal ischemia (RI group, *n* = 6) (Brown et al., 2019) or transient unilateral renal ischemia followed by delayed contralateral nephrectomy (RI‐DCN group, *n* = 5; banked samples also used in a previous study (Grimes et al., 2022)). Healthy cats (*n* = 8) were participants of unrelated terminal studies for which normal kidney function and histopathology were documented as part of a previous study (Lourenço et al., 2020), whose data and samples included in the current study were collected at a single time point (at the time of euthanasia). All activities related to the original studies were approved by the University of Georgia's Institutional Animal Care and Use Committee.

**FIGURE 1:**
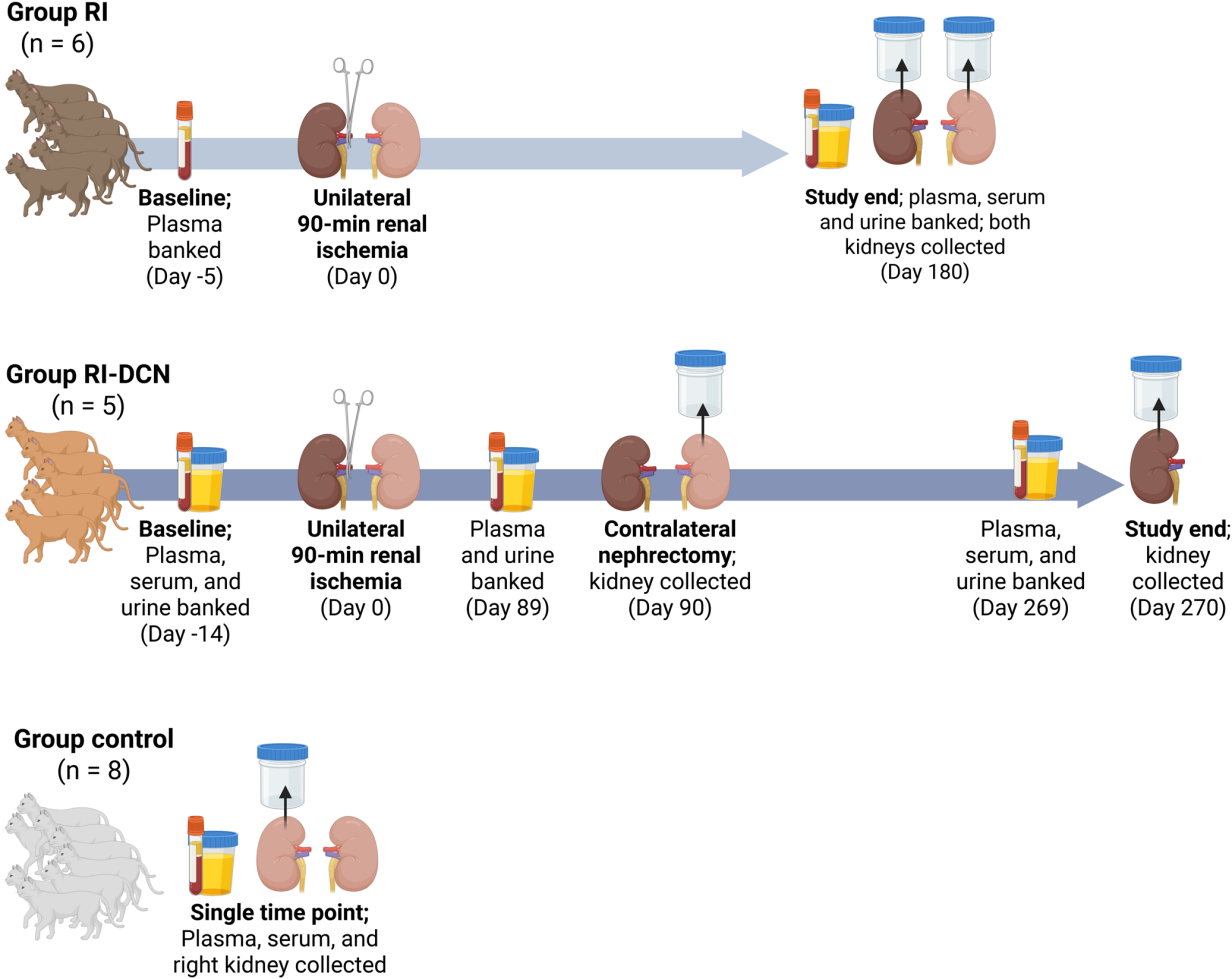
Overview of original studies from which the groups evaluated in the present study were generated: Unilateral renal ischemia (RI, *n* = 6; top panel); unilateral renal ischemia followed by delayed contralateral nephrectomy (RI‐DCN, *n* = 5; middle panel); and the healthy control group (*n* = 8; bottom panel). Lourenço, B. (nd). Circulating and intrarenal RA(A)S study. Created in BioRender. https://BioRender.com/wf37jn7.

A general overview of the original CKD model studies and data and sample collection for CKD and healthy cats is presented in Figure 1. Briefly, cats of the RI group underwent general anesthesia for temporary cross‐clamping of the right renal artery and vein for 90 min while the contralateral (left) kidney was undisturbed (Brown et al., 2019). Six months later, both kidneys were collected at the end of that study. Cats of the RI‐DCN group underwent temporary unilateral right renal ischemia using the same technique. Three months later, the contralateral (left) kidney was removed and collected. At study end, 6 months after contralateral nephrectomy (9 months after the initial surgery), the previously ischemic right kidney was collected for purposes of that previous study. Blood and urine samples and arterial blood pressure measurements were collected at several time points in both groups, as specified in Figure 1, Table 1.

**TABLE 1:** Available information and samples for each study time point in the 19 purpose‐bred adult cats used in the present study.

Group	*n*	Timepoint (Day 0 = RI)	Plasma‐PRA	Serum‐cRAAS	Kidney tissue	Urine‐UACR	Blood pressure
RI	6	Baseline (Day ‐5)	6	‐	‐	‐	6 (ind)
		Study end (Day 180)	6	6	6	3	6 (ind)
RI‐DCN	5	Baseline (Day ‐14)	5	4	‐	5	3 (dir)
		Day 89	5	‐	5	5	4 (dir)
		Day 93^a^	1	‐	1	1	‐
		Day 97	4	‐	‐	‐	3 (dir)
		Study end (Day 269)	4	4	4	4	2
Control	8	Single time point (euthanasia)	8	6	8	7	‐

Abbreviations: cRAAS, circulating renin‐angiotensin‐aldosterone system; dir, directly measured; ind, indirectly measured; PRA, plasma renin activity; RI, unilateral renal ischemia; RI‐DCN, unilateral renal ischemia followed by delayed contralateral nephrectomy; UACR, urinary protein‐to‐creatinine ratio; ‐, not applicable.

^a^ One cat was euthanized on Day 93 after reaching the original study's predetermined endpoint for humane euthanasia.

During the original studies, cats in the RI and control groups were fed a dry maintenance cat food (Purina Cat Chow Premium Cat Food, Nestlé Purina) with a sodium content of 153–165 mg/100 kcal (Lourenço et al., 2020). Cats in the RI‐DCN group were also fed this diet before and for 2 weeks after RI, after which they were fed a different diet (Hill's® Science Diet® Adult 7+ Active Longevity Original Cat Dry Food, Hill's Pet Nutrition, Inc.) with a lower sodium content (66 mg/100 kcal) for the remainder of the study. Diet had been changed in that RI‐DCN study so that dietary sodium content more accurately matched that of client‐owned cats with spontaneous CKD, who are typically fed a lower‐sodium, clinical renal diet in keeping with current guidelines for management of chronic kidney disease in cats (International Renal Interest Society, 2023). Husbandry conditions were otherwise similar for all cats.

### 2.2 Sample collection

Renal tissues were collected at the time points described in Figure 1 and Table 1, with samples taken from both kidneys in cats of the RI and RI‐DCN groups and from the right kidney in cats of the control group (Lourenço et al., 2020). All renal tissues were collected and processed during surgery or within 1 h of euthanasia. Collected kidneys were sectioned longitudinally. One‐half of each kidney was immediately minced and placed in RNA stabilization solution (RNA*later* stabilization solution, catalog number 76106, QIAGEN; Valencia, CA, USA), and the remaining portion was placed in neutral‐buffered 10% formalin. Following overnight incubation at 4°C, tissues were removed from the RNA stabilization solution, homogenized with a mortar and pestle, divided into 30–100 mg aliquots, and stored at −80°C.

Venous blood for plasma samples was collected into tubes containing EDTA, immediately placed on ice, and centrifuged under refrigeration at 4°C. Venous blood for serum samples was collected into glass tubes with no additive, allowed to clot, and centrifuged. When available, urine was collected by cystocentesis or from litter boxes containing non‐absorbent plastic beads, and placed into glass tubes. All biological samples were aliquoted and stored at −80°C until analysis.

### 2.3 Clinical parameter measurements

Cats' general health and kidney function were evaluated by hematologic, serum biochemical, and urinary (i.e., urinalysis and urinary protein‐to‐creatinine ratio) analyses, conducted by the Clinical Pathology Laboratory of the College of Veterinary Medicine, University of Georgia (Athens, GA, USA).

For cats in the RI group, indirect systemic arterial blood pressure (BP) was measured in awake, unsedated cats using Doppler sphygmomanometry, following guidelines of the American College of Veterinary Internal Medicine (Acierno et al., 2018). For cats in the RI‐DCN group, direct femoral arterial BP was measured in awake, unsedated cats using surgically implanted telemetric BP‐sensing devices (Data Sciences International; St. Paul, MN, USA), as previously described (De Lombaert et al., 2023). These devices were implanted prior to the start of the original study. Briefly, these devices measure and transmit direct BP data as a radio signal to radiotelemetry receivers (Model RMC‐1, Data Sciences International; St. Paul, MN, USA), which route these data to a dedicated computer‐based acquisition system (Ponemah V 5.2, Data Sciences International; St. Paul, MN, USA). During BP measurement periods, cats were undisturbed in their vivarium, and their usual cages were outfitted with radiotelemetric receivers. Data recorded in the first 30 and last 5 min of the measurement period (i.e., when researchers entered the room to begin or end the session) were excluded from analysis. The remaining values, generated by averaging measurements taken for 10 s every 22 s, were averaged to generate a single session value for systolic BP, which was used in analyses.

### 2.4 Measurements of RA(A)S markers

Serum, plasma, and renal tissue homogenates were shipped on dry ice to a commercial laboratory, where RA(A)S markers were measured (Attoquant Diagnostics GmbH, Vienna, Austria). Frozen renal tissue samples (80–100 mg) were homogenized using a mortar and pestle under liquid nitrogen. The frozen tissue powder was dissolved to 100 mg/mL in 6 mol/L aqueous guanidinium chloride (catalog number 50940, Sigma‐Aldrich; Steinheim, Germany) supplemented with 1% (v/v) trifluoroacetic acid (catalog number T6508, Sigma‐Aldrich; Steinheim, Germany) by cooled sonication using a 2 mm microtip (2 mm microtip, Sonics and Materials, Newton, New Jersey). Stable isotope‐labeled internal standards for individual angiotensin metabolites were added to tissue homogenates at 200 pg/mL. All serum and renal tissue samples were stored at −80°C until analysis.

Equilibrium concentrations of angiotensin I, II, III, IV, 1–5, 1–7, and aldosterone were measured in serum samples, and equilibrium concentrations of angiotensin I, II, III, and IV were measured in renal tissue homogenates, using a previously described and validated (van Rooyen et al., 2016; Domenig et al., 2016; Guo, Poglitsch, McWhinney, et al., 2020; Haschke et al., 2013; Huh et al., 2021) liquid chromatography–tandem mass spectrometry (LC–MS/MS) protocol (RAS‐Fingerprint™).

Briefly, serum samples were equilibrated at 37°C for 1 h, followed by stabilization through addition of an enzyme inhibitor cocktail. The samples were then spiked with stable isotope‐labeled internal standards at 200 pg/mL for each analyte and underwent C‐18‐based solid‐phase‐extraction. Processed serum and tissue homogenate samples then underwent LC–MS/MS analysis using a reversed‐phase analytical column operating in line with a Xevo TQS triple quadruple mass spectrometer (Waters; Milford, MA, USA).

Surrogate markers for serum enzyme activity and adrenal responsiveness to Ang II were calculated using measured equilibrium‐based serum concentrations of angiotensin peptides and aldosterone, as previously reported:

1. PRA‐S (surrogate for plasma renin activity, pM) = [Ang I]_serum_ + [Ang II]_serum_ (Pavo et al., 2018)
2. ACE‐S (surrogate for angiotensin‐converting enzyme (ACE) activity, pM/pM) = [Ang II]_serum_/[Ang I]_serum_ (Guo, Poglitsch, Cowley, et al., 2020)
3. AA2 (surrogate measure of adrenal responsiveness to Ang II, pM/pM) = [aldosterone]_serum_/[Ang II]_serum_ (Burrello et al., 2020)

Plasma renin activity (PRA) was measured on EDTA‐preserved plasma by the same commercial laboratory using a previously described LC–MS/MS‐based method (Simko et al., 2018). Briefly, plasma was diluted in an angiotensin I‐generating buffer containing 5 μM ethylenediaminetetraacetic acid, 20 μM Z‐Pro‐Prolinal, 1 mM 4‐(2‐Aminoethyl)benzene‐sulfonyl fluoride hydrochloride, and 10 μM aminopeptidase inhibitor in phosphate‐buffered saline. Mixtures were then split into two aliquots. One was placed on ice and served as the baseline control. The other was incubated at 37°C for 1 h, stabilized with inhibitor cocktail, then spiked with a stable isotope‐labeled internal standard for angiotensin I at a concentration of 200 pg/mL. The angiotensin I concentration was then quantified using the same LC–MS/MS technique described above. PRA was calculated by subtracting the angiotensin I formation in the incubated aliquot from the baseline control and was expressed as (ng/mL)/h.

Urine was shipped overnight on dry ice to a separate external laboratory (Michigan State University Diagnostic Laboratory, Lansing, MI, USA), where urinary aldosterone‐to‐creatinine ratio (UACR) was determined. Urinary aldosterone was measured with a commercial radioimmunoassay kit (Active Aldosterone RIA, DSL8600, Beckman Coulter; Fullerton, CA, USA). Urinary creatinine was measured using the modified Jaffe method. The UACR (pmol/mmol) was calculated as urinary aldosterone concentration (pmol/L) divided by the urinary creatinine concentration (mmol/L).

### 2.5 Renal gene transcription quantification

Reverse‐transcription and quantitative polymerase chain reaction (PCR) were performed in‐house to quantify transcript levels of angiotensinogen (*AGT*), renin (*REN*), angiotensin‐converting enzyme (*ACE*), and angiotensin II type 1 receptor (*AT1R*) in renal tissue homogenates using a previously described protocol employing feline gene‐specific primers (Lourenço et al., 2022). The reader is directed to reference (Lourenço et al., 2022) for a detailed description of the methods used, including primers, which were obtained from Integrated DNA Technologies, Inc., Coralville, IA. Briefly, RNA was extracted from tissue homogenates using a commercial kit (RNeasy Plus Mini Kit, catalog number 74192, QIAGEN; Valencia, CA, USA) and its integrity checked with a spectrophotometer (NanoDrop, Thermo Fisher Scientific; Waltham, MA, USA) and visualization of 18S and 20S ribosomal bands on agarose gels. RNA samples were reverse transcribed into cDNA using a commercial kit (SuperScript IV VILO Master Mix, catalog number 11756050, Invitrogen; Carlsbad, CA, USA) and quantitative PCR was conducted with an automated cycler (CFX96, Bio‐Rad Laboratories; Hercules, CA, USA). All reactions were performed in triplicate, and mean values were used for further analyses.

Transcript levels of the target genes *ACE*, AGT, *AT1R*, and *REN* were normalized to those of two reference genes, *GAPDH* and ribosomal protein S7 (*RPS7*), using the GENorm method (Vandesompele et al., 2002). After normalization, transcript levels were scaled to those of the lowest sample for each target gene.

Quantitative PCR products were confirmed on 1.2% agarose gels. Amplicons were cloned into a vector system (PGEM‐T easy vector, Promega, Madison, WI) and sequenced bi‐directionally by chain termination (Molecular Cloning Laboratories, San Francisco, California). The resulting sequences were confirmed using the National Center for Biotechnology Information Basic Local Alignment Search Tool against the feline genome (Ye et al., 2012).

### 2.6 Statistical analysis

Statistical analyses were performed with R (version 4.3.3). Linear mixed models were built with the package lme4 (version 1.1–35.2), and pairwise comparisons between groups were conducted with the package emmeans (version 1.10.1). All package parameters were left as default unless otherwise specified. *p*‐values <0.05 were considered statistically significant.

Distribution of values for continuous variables was examined for normality using the Shapiro–Wilk test. For clinical parameters, normally distributed data are presented as mean ± SD and compared between groups using a one‐way ANOVA with pairwise comparisons adjusted using the Bonferroni method. Non‐normally distributed data are presented as median (range) and compared between groups using the Kruskal‐Wallis test with subsequent pairwise comparisons adjusted using the Bonferroni method. For the purposes of statistical analyses, urine specific gravity values >1.060 were assigned a value of 1.061 and angiotensin peptide concentrations less than the lower limit of quantification were assigned a value of the lower limit divided by 2 (Hornung & Reed, 1990). For the following regression and correlation analysis, RA(A)S markers, including PRA, UACR, gene transcription levels, angiotensin peptide, and circulating aldosterone concentrations, and calculated RAAS surrogate indices, were all log‐transformed to improve the approximation of normal distribution.

Linear mixed models were used to compare BP, PRA, UACR, and circulating RA(A)S markers between cats of the healthy control and CKD (i.e., RI and RI‐DCN) groups and between different study time points. In addition, linear mixed models were used to compare intrarenal RAS markers (i.e., renal angiotensin peptide concentration and transcript levels of RAS‐related genes) between ischemic right kidneys or non‐ischemic left kidneys (for RI and RI‐DCN groups) and undisturbed right kidneys of the control group. Group (healthy control, RI, RI‐DCN), kidney (ischemic or non‐ischemic kidney of RI and RI‐DCN groups, right kidney of control group), or study timepoint were set as fixed effects; individual cats were set as random effects; and the parameter of interest (i.e., BP, PRA, UACR, or RA[A]S marker) were set as the outcome. Once the models were built, each study time point was compared to baseline; each CKD group was compared to the healthy control group; and each kidney of each of the two CKD groups was compared with the right kidneys of the healthy control group. Adjustments for multiple comparisons were made using Tukey's method.

Correlations between markers of circulating RAAS activity (PRA, UACR, serum aldosterone), intrarenal RAS markers, and serum creatinine as a surrogate of renal function were analyzed using Pearson's correlation analysis. Data were used if biological samples from the individual cat were collected within 24 h of one another, and parameter values across all groups were combined. Parameters found to be significantly correlated with serum creatinine (as a surrogate for kidney function) or intrarenal angiotensin II concentration (as a surrogate for intrarenal RAS activity) were then evaluated using multiple linear regression models.

## 3 RESULTS

### 3.1 Sample availability

Biological samples that were available for evaluation in the present study are summarized in Table 1. One cat in the RI‐DCN group met the original study's predetermined endpoint for humane euthanasia for compassionate care and was euthanized on study Day 93 (i.e., 3 days after nephrectomy) due to severe azotemia (i.e., serum creatinine concentration >8 mg/dL); data from all time points prior to euthanasia were included in the analyses.

### 3.2 Clinical data

Demographic and clinical information by study group is summarized in Table 2. At study end, serum creatinine concentration was significantly different among all three groups, with cats in the RI group exhibiting milder functional impairment than those in the RI‐DCN group. Serum creatinine concentration was within the laboratory's reference interval for most cats in the RI group at study end. Serum sodium and chloride were higher, while potassium was lower, in the RI group.

**TABLE 2:** Demographic and clinical information for the 19 purpose‐bred adult cats used in the present study.

	Control (*n* = 8)	RI (*n* = 6)		RI‐DCN (*n* = 5)			*p*‐value (study end comparisons)
Study time point	Single time point (euthanasia)	Baseline	Study end (Day 180)	Baseline	Before contralateral nephrectomy (Day 89)	Study end (Day 270)*	
Age (years)	0.7 (0.6–0.8)^a^	0.9 (0.8–1.1)	1.4 (1.3–1.6)^b^	1.7 (0.5–1.7)	1.9 (0.7–1.9)	2.4 (1.2–2.4)^c^	0.001
Sex (*n*)							
Female spayed	0	6		0		0	‐
Female intact	8	0		0		0	
Male neutered	0	0		5		4	
Body weight (kg)	3.6 (2.3–4.4)^a^	3.0 (2.6–4.1)	4.7 (3.9–5.3)^b^	6.6 (5.6–7)	6.3 (5.5–6.9)	5.8 (4.3–7.0)^b^	0.003
sCr (mg/dL) Ref. Int., 0.6–1.8	1.0 (0.8–1.2)^a^	1.1 (0.9–1.2)	1.4 (1.3–1.6)^b^	1.1 (1.0–1.4)	1.3 (1.1–1.3)	1.6 (1.6–9.3)^c^	0.001
SUN (mg/dL) Ref. Int., 21–36	27 (23–34)	24 (21–30)	23.5 (22–22)	19 (17–21)	np	30.5 (23–87)	0.094
SDMA (μg/dL) Ref. Int., 0–14	12 (9–16)	np	8 (6–10)	np	np	np	0.011
Serum sodium (mmol/L) Ref. Int., 147–154	152 (149–152)^a^	np	158 (157–159)^b^	152 (148–154)	np	152 (149–153)^a^	0.004
Serum potassium (mmol/L) Ref. Int., 3.2–5.6	4.20 (3.71–4.59)^ab^	np	4.45 (4.31–4.69)^a^	4.30 (4.01–4.98)	np	4.00 (2.93–4.20)^b^	0.040
Serum chloride (mmol/L) Ref. Int., 110–122	112 (111–116)^a^	np	119 (117–120)^b^	114 (110–119)	np	117 (107–119)^ab^	0.012
Serum phosphate (mmol/L) Ref. Int., 2.9–5.8	5.80 (5.41–6.57)	np	6.10 (5.91–6.77)	4.70 (3.74–5.28)	np	4.65 (4.21–10.0)	0.177
Serum bicarbonate (mmol/L) Ref. Int., 15–23	20.5 (16.0–22.0)	np	20.0 (19.0–23.0)	21.0 (19.0–22.9)	np	17.5 (15.0–21.0)	0.370
USG	1.053 (1.037–1.061)^a^	1.048 (1.046–1.052)	1.056 (1.041–1.059)^a^	1.053 (1.047–1.058)	1.055 (1.047–1.059)	1.032 (1.012–1.047)^b^	0.018
UPC Ref. Int., 0.0–0.4	0.14 (0.1–0.2)^a^	0.14 (0.08–0.22)	0.10 (0.05–0.11)^b^	0.12 (0.07–0.75)	np	0.14 (0.08–0.31)^ab^	0.023
Histopathology	Normal	np	IK: tubulointerstitial inflammation and fibrosis NIK: Normal	np	NIK: normal	IK: tubulointerstitial inflammation and fibrosis	‐

*Note*: Data are presented as median (range).

Abbreviations: IK, ischemic kidney; NIK, non‐ischemic kidney; np, not performed; RI, unilateral renal ischemia; RI‐DCN, unilateral renal ischemia followed by delayed contralateral nephrectomy; sCr, serum creatinine concentration; SDMA, serum symmetric dimethylarginine concentration; SUN, serum urea nitrogen; UPC, urinary protein‐to‐creatinine ratio; USG, urine specific gravity.

* Data available for only *n* = 4 cats at study end in RI‐DCN group. Values with different superscripts are significantly different at study end in pairwise comparison after Bonferroni adjustment, with an overall significant level of 0.05.

^a–c^ Within each row, values with different superscript letters are significantly different (*p* < 0.05).

### 3.3 PRA, UACR, and BP in cats with surgically induced CKD

**FIGURE 2:**
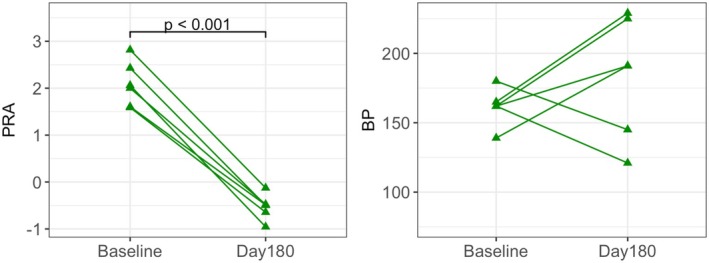
Dot plots of plasma renin activity (PRA; log‐transformed) and indirect systolic arterial blood pressure (BP; mmHg) before and 180 days after surgically induced ischemic renal injury in cats of the RI group (*n* = 6). Data from the same individual are connected by solid lines.

**FIGURE 3:**
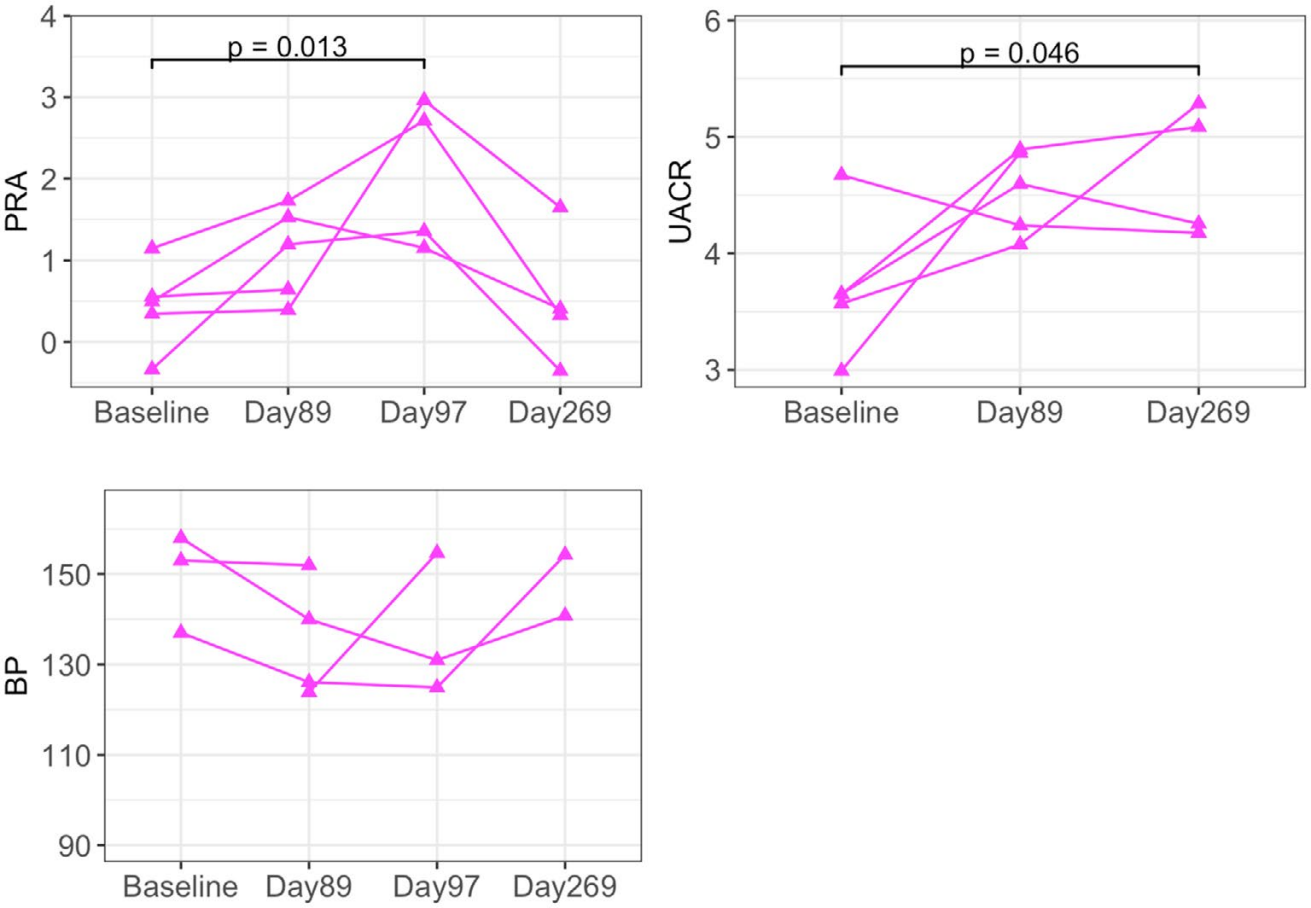
Dot plots of log‐transformed plasma renin activity (PRA), log‐transformed urinary aldosterone‐to‐creatinine ratio (UACR), and direct systolic arterial blood pressure (BP; mmHg) over the study period in cats of the RI‐DCN group (*n* = 5). Data from the same individual are connected by solid lines.

Values for BP, PRA, and UACR (the latter for the RI‐DCN group only) are depicted in Figures 2 and 3 for the RI and RI‐DCN groups, respectively. Direct BP measurement was not successful in all cats and all time points for the RI‐DCN group due to equipment failures. A summary of the regression models for these data is presented in Table 3.

**TABLE 3:** Results of linear mixed models for log‐transformed plasma renin activity, log‐transformed urinary aldosterone‐to‐creatinine ratio, and systemic arterial blood pressure.

	Estimated marginal means	*p*‐value
Plasma renin activity		
Group RI		
Day 180‐Baseline	−2.614	<0.001
Group RI‐DCN		
Day 89‐Baseline	0.657	0.324
Day 97‐Baseline	1.594	0.013
Day 269‐Baseline	0.056	0.994
Urinary aldosterone‐to‐creatinine ratio		
Group RI‐DCN		
Day 89‐Baseline	0.827	0.072
Day 269‐Baseline	0.993	0.046
Systolic arterial blood pressure (mmHg)		
Group RI		
Day 180‐Baseline	22.000	0.285
Group RI‐DCN		
Day 89‐Baseline	−13.884	0.457
Day 97‐Baseline	−12.505	0.583
Day 269‐Baseline	−1.826	0.992

Abbreviations: RI, unilateral renal ischemia; RI‐DCN, unilateral renal ischemia followed by delayed contralateral nephrectomy.

In the RI group, PRA was significantly lower on Day 180 compared to baseline (*p* < 0.001). In the RI‐DCN group, a significant increase in PRA was noted on Day 97 compared to baseline (*p* = 0.013), with return on Day 269 to levels not significantly different from baseline.

### 3.4 Group comparisons of circulating RAAS markers

**FIGURE 4:**
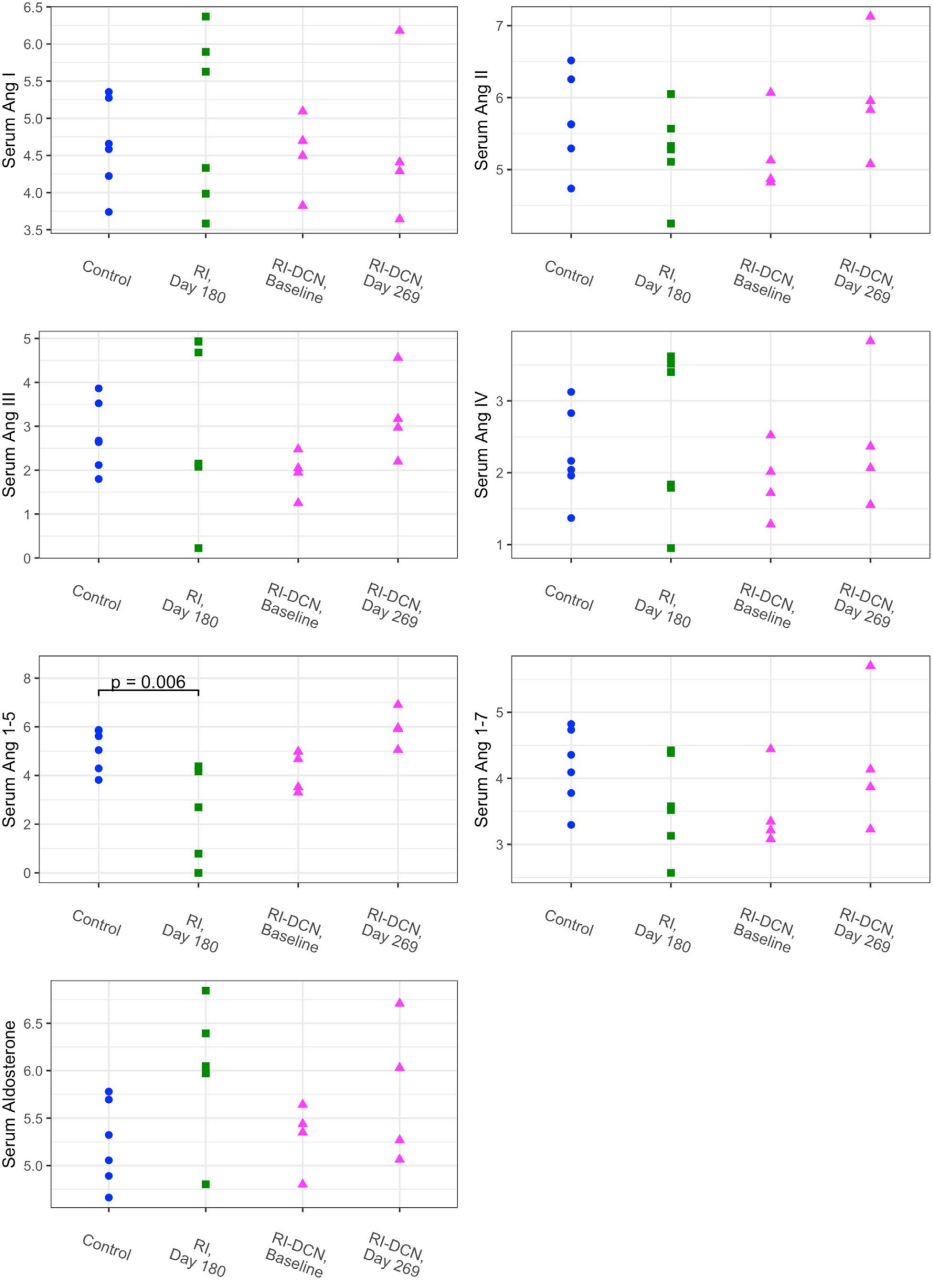
Dot plots of log‐transformed serum angiotensin peptide and aldosterone equilibrium concentrations by study group. Data from the control, RI, and RI‐DCN group are plotted as blue circles, green squares, and magenta triangles, respectively. Ang, angiotensin; RI‐DCN, unilateral renal ischemia followed by delayed contralateral nephrectomy; RI, unilateral renal ischemia.

**FIGURE 5:**
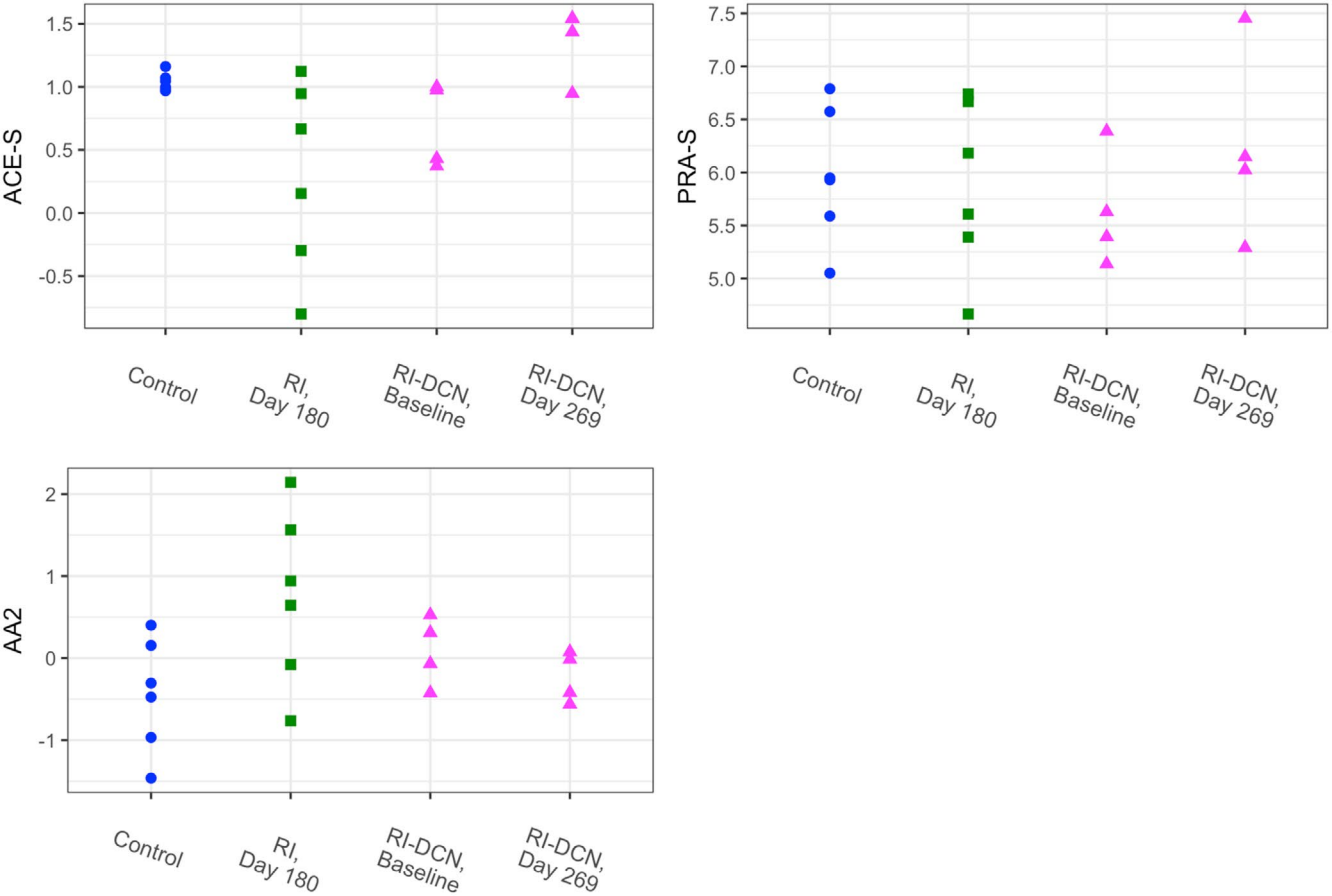
Dot plots of log‐transformed circulating renin‐angiotensin‐aldosterone system surrogate indices by study group. Data from the control, RI, and RI‐DCN group are plotted as blue circles, green squares, and magenta triangles, respectively. AA2, aldosterone‐to‐angiotensin II ratio; ACE‐S, surrogate for angiotensin‐converting enzyme activity; PRA‐S, surrogate for plasma renin activity; RI‐DCN, unilateral renal ischemia followed by delayed contralateral nephrectomy; RI, unilateral renal ischemia.

Angiotensin peptide and aldosterone concentrations and RAAS surrogate indices are presented by group in Figures 4 and 5, respectively. Information from relevant regression models is summarized in Table S1. The only circulating angiotensin peptide concentration that differed significantly between healthy controls and one of the CKD groups was that of Ang 1–5, which was lower in cats of the RI group on Day 180 (*p* = 0.004).

### 3.5 Group comparisons of intrarenal RAS markers

**FIGURE 6:**
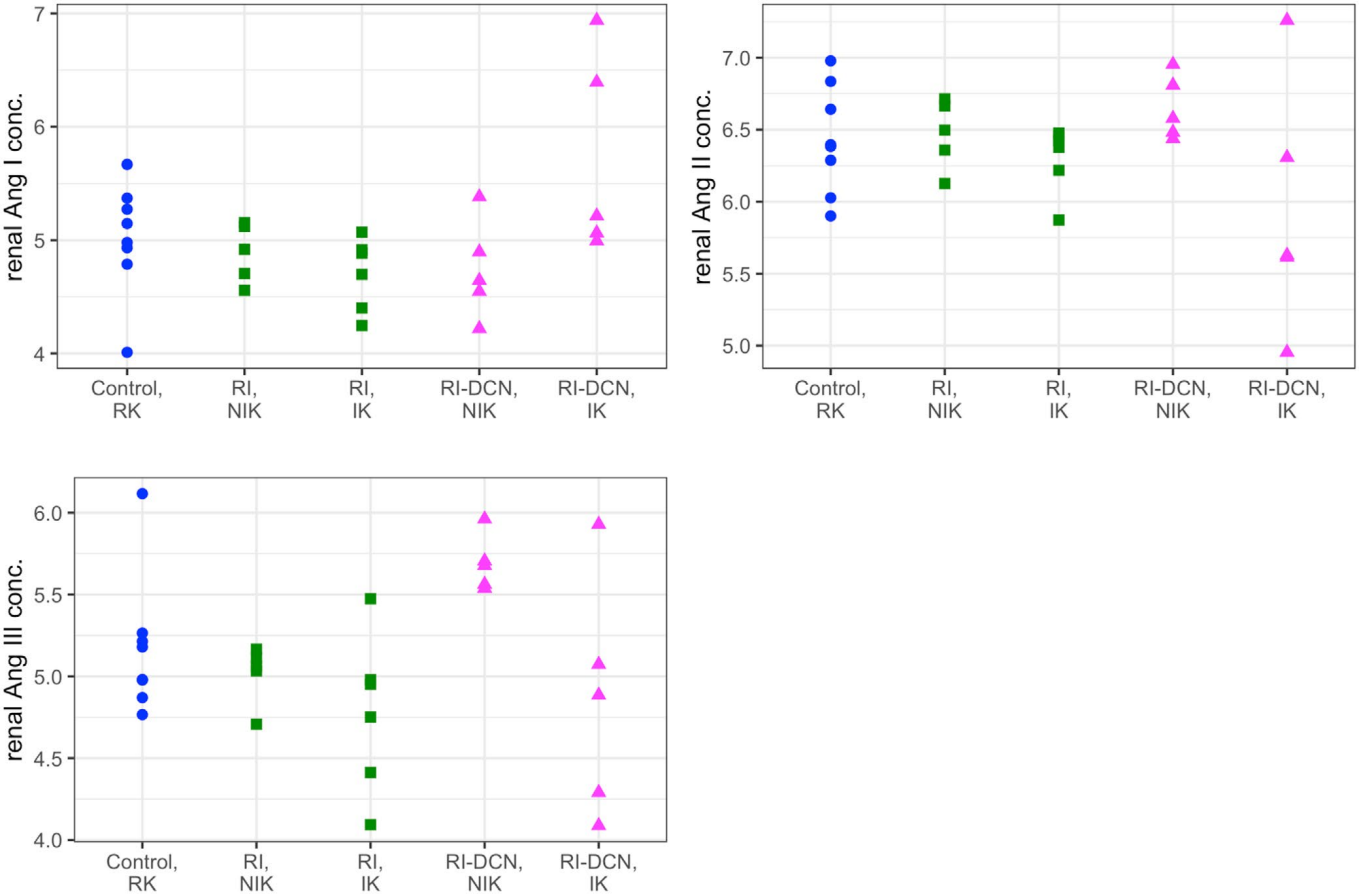
Dot plots of log‐transformed renal tissular angiotensin peptide equilibrium concentrations by group and kidney. Data from the control, RI, and RI‐DCN group are plotted as blue circles, green squares, and magenta triangles, respectively. Ang, angiotensin; IK, ischemic kidney; NIK, non‐ischemic kidney; RI, unilateral renal ischemia; RI‐DCN, unilateral renal ischemia followed by delayed contralateral nephrectomy; RK, right kidney.

Angiotensin peptide concentrations in ischemic or non‐ischemic kidneys of both CKD model groups and in the right kidneys of the healthy control group are presented in Figure 6. Information from relevant regression models is summarized in Table S2. No measured peptide concentration differed significantly between either kidney of the two CKD model groups and the kidneys of the healthy control group.

**FIGURE 7:**
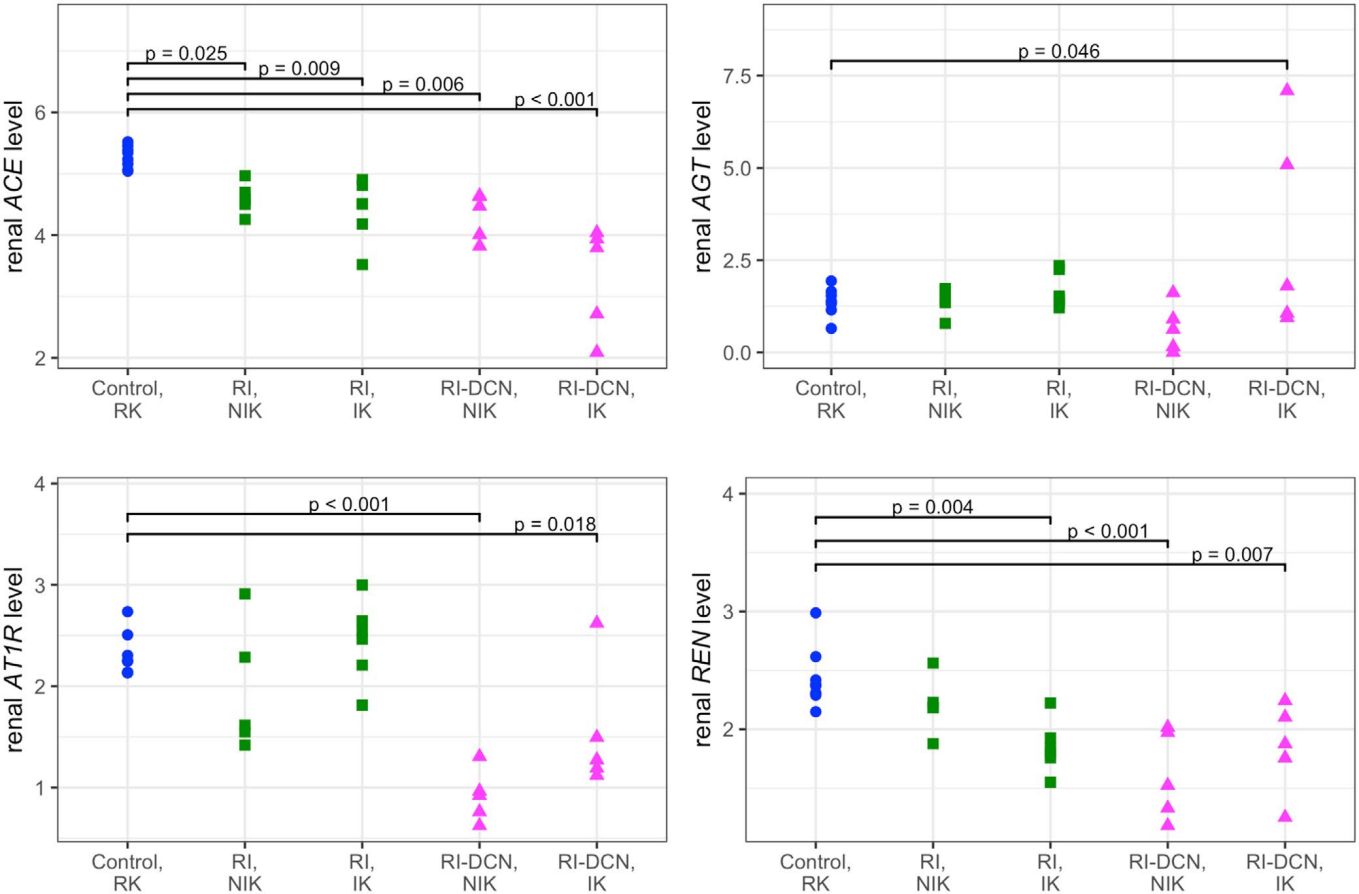
Dot plots of log‐transformed renal tissular gene transcript levels by group and kidney. Data from the control, RI, and RI‐DCN group are plotted as blue circles, green squares, and magenta triangles, respectively. *ACE*, angiotensin‐converting enzyme; *AGT*, angiotensinogen; *AT1R*, angiotensin type‐1 receptor; IK, ischemic kidney; NIK, non‐ischemic kidney; *REN*, renin; RI, unilateral renal ischemia; RI‐DCN, unilateral renal ischemia followed by delayed contralateral nephrectomy; RK, right kidney.

Renal transcription levels of *ACE*, *AGT*, *AT1R*, and *REN* are presented in Figure 7. Information from relevant regression models is summarized in Table S2. Compared to the healthy control group, both ischemic and non‐ischemic kidneys from the RI‐DCN group had significantly lower transcript levels of *ACE* (*p* < 0.001 and *p* = 0.006, respectively), *AT1R* (*p* = 0.018 and *p* < 0.001, respectively) and *REN* (*p* = 0.007 and *p* < 0.001, respectively), while ischemic kidneys of the RI‐DCN group had significantly higher transcript levels of *AGT* (*p* = 0.046). Compared to the healthy control group, ischemic kidneys from cats of the RI group had significantly lower transcript levels of *REN* (*p* = 0.004). Within‐group comparisons of peptide concentrations and gene transcript levels between the ischemic and non‐ischemic kidneys were performed using paired *t*‐tests, with results shown in Figure S1.

### 3.6 Correlations between circulating and intrarenal RA(A)S markers

**FIGURE 8:**
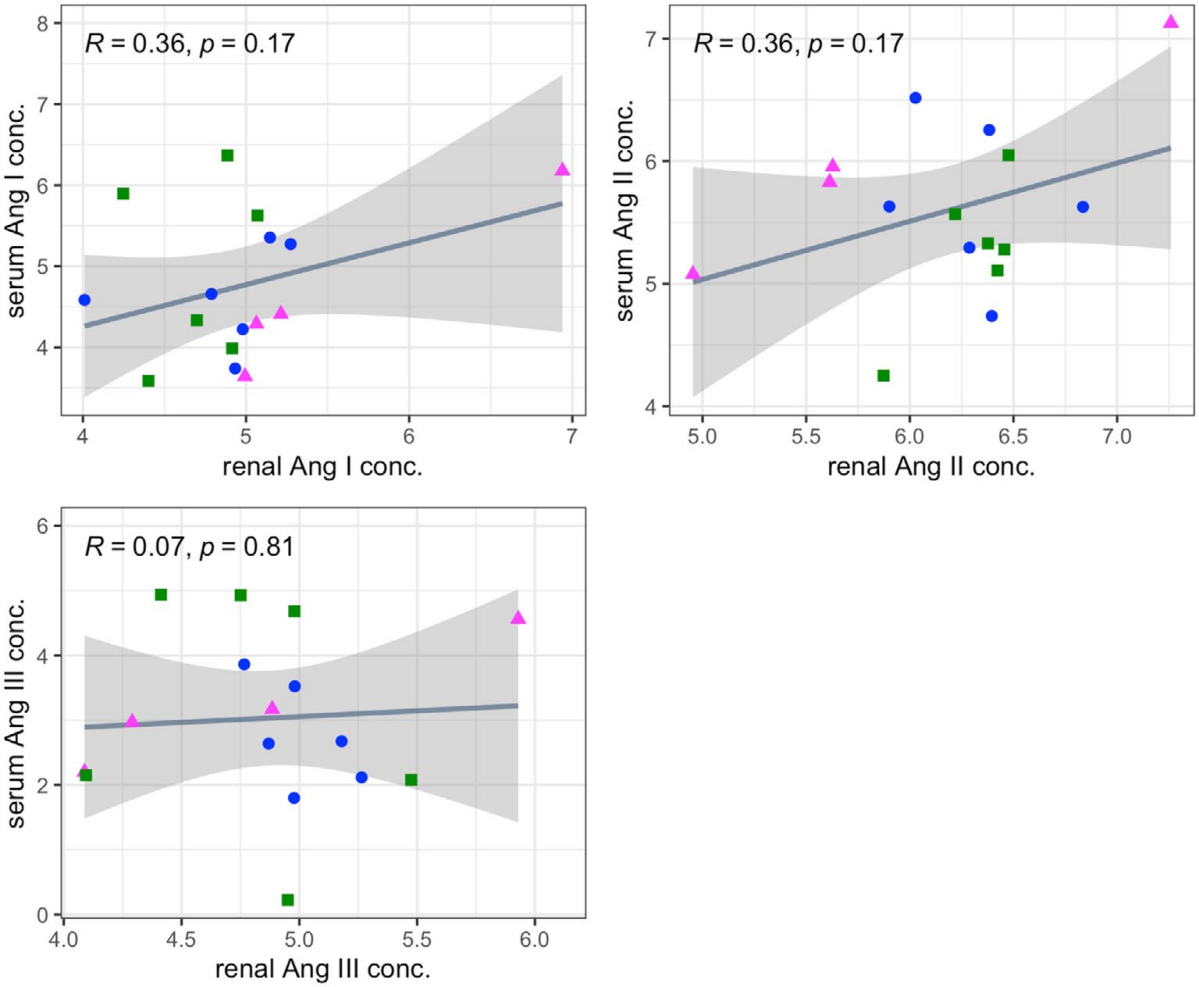
Scatterplots of log‐transformed equilibrium concentrations of angiotensin I, II, or III in serum versus renal tissue, with lines of best fit and 95% confidence intervals (shaded areas) displayed. Correlation coefficient (*R*) and *p*‐values were calculated using Pearson's correlation analysis. Data from the control, RI, and RI‐DCN group are plotted as blue circles, green squares, and magenta triangles, respectively. Ang, angiotensin; RI‐DCN, unilateral renal ischemia followed by delayed contralateral nephrectomy; RI, unilateral renal ischemia.

Results of correlation analysis involving all parameters are presented in Table S3. Circulating concentrations of angiotensin I, II, and III were not significantly correlated with their renal tissular concentrations (Figure 8).

**FIGURE 9:**
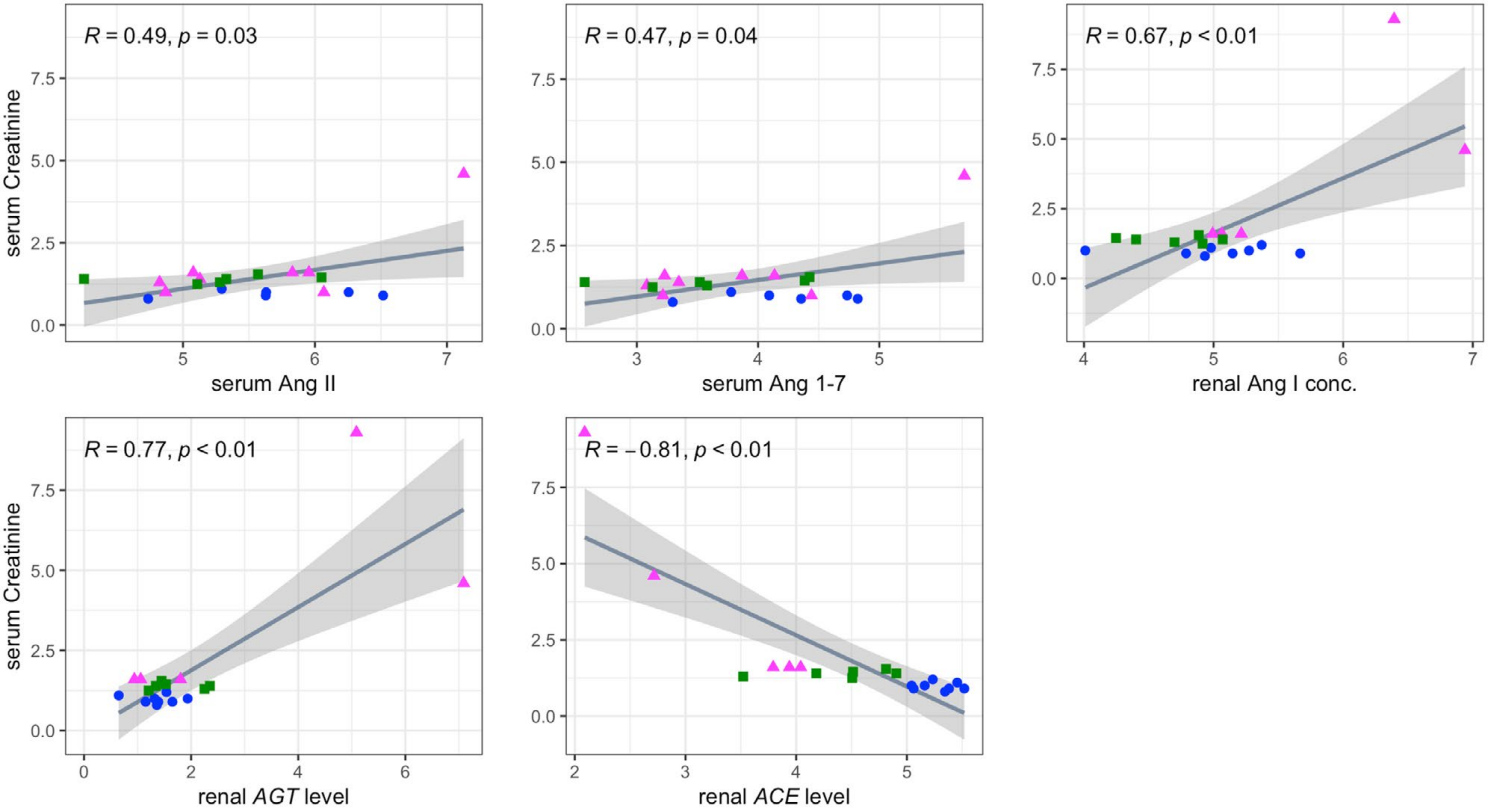
Scatterplots of circulating and intrarenal renin‐angiotensin(‐aldosterone) system parameters versus serum creatinine concentration. Lines of best fit and 95% confidence intervals (shaded areas) are displayed. *R* and *p*‐values were calculated using Pearson's correlation analysis. Data from the control, RI, and RI‐DCN group are plotted as blue circles, green squares, and red triangles, respectively. *ACE*, angiotensin‐converting enzyme; *AGT*, angiotensinogen.

We also examined possible correlations between circulating or renal RA(A)S markers and serum creatinine concentration, the latter an endogenous marker of renal functional impairment and CKD severity. Serum concentrations of angiotensin II and angiotensin 1–7, intrarenal concentrations of angiotensin I, and renal transcript levels of *AGT* were all significantly positively correlated with serum creatinine concentration. Conversely, renal transcript levels of *ACE* were significantly negatively correlated with serum creatinine concentration (Figure 9). Based on the results of multiple linear regression analysis, intrarenal concentrations of angiotensin I and renal transcript levels of *AGT* and *ACE* were independently correlated with serum creatinine concentration (Table S4).

**FIGURE 10:**
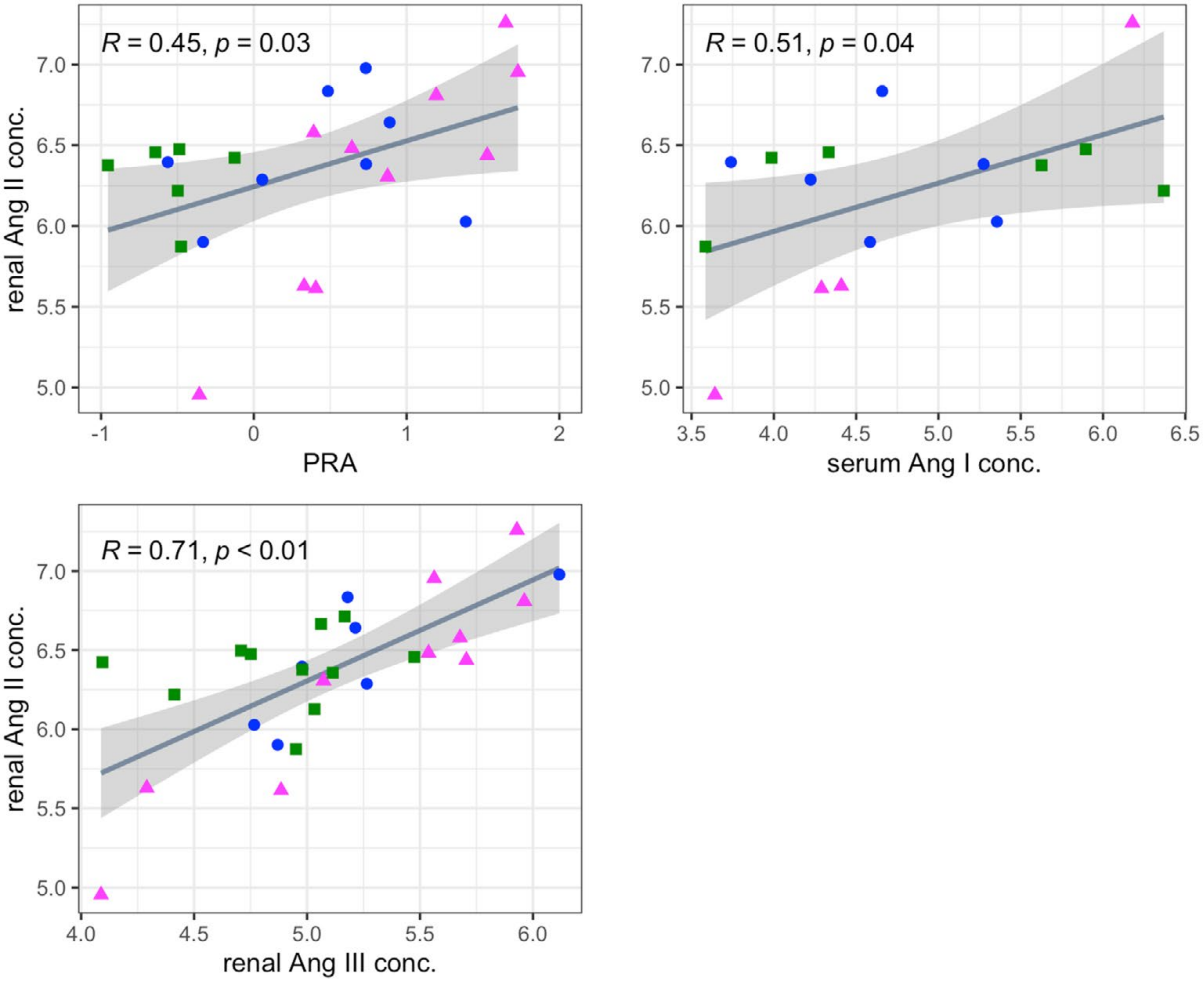
Scatterplots of circulating and intrarenal renin‐angiotensin‐aldosterone system parameters versus intrarenal angiotensin II concentrations. Lines of best fit and 95% confidence intervals (shaded areas) are displayed. Correlation coefficient (*R*) and *p*‐values were calculated using Pearson's correlation analysis. Data from the control, RI, and RI‐DCN group are plotted as blue circles, green squares, and magenta triangles, respectively. Ang, angiotensin; PRA, plasma renin activity.

Similarly, we investigated possible correlations between the same parameters and intrarenal angiotensin II concentrations. Serum angiotensin I concentrations, PRA, and intrarenal angiotensin III concentrations were significantly positively correlated with intrarenal angiotensin II (Figure 10). Based on the results of multiple linear regression analysis, only intrarenal Ang III was independently correlated with intrarenal Ang II concentration (Table S4).

## 4 DISCUSSION

In the study reported here, circulating and intrarenal RA(A)S markers were compared within and between cats with one of two models of surgically induced CKD and healthy cats. Compared to healthy cats, we found that kidney transcript levels of *AGT* were greater in cats with the more severe of our two CKD models, while transcript levels of *ACE*, *AT1R*, and *REN* were lower in one or both models. Neither circulating nor intrarenal angiotensin peptide concentrations were significantly different between healthy cats and those with induced CKD, except for circulating angiotensin 1–5, which was lower in one of the CKD model groups. Intrarenal angiotensin peptides were not significantly correlated with their circulating counterparts.

When PRA, UACR, and BP were evaluated longitudinally, cats of the RI‐DCN group experienced significantly increased UACR at study end compared to baseline. While the same cats had significantly increased PRA 97 days after unilateral renal ischemia, this activity had returned to baseline levels when measured at study end. Meanwhile, 180 days after renal ischemia, cats of the RI group had decreased PRA compared to baseline.

PRA is known to decrease as individuals age. This is true for both healthy cats (Javadi et al., 2004) and people (Dillon & Ryness, 1975). In the present study, most cats in the RI group and some cats in the RI‐DCN group transitioned from kitten to the young adult life stage (Quimby et al., 2021) over the course of the study period. In human beings, similar life stage transitions are accompanied by dramatic PRA decreases (Dillon & Ryness, 1975). The general decrease in PRA over time in cats of the RI group might have been an effect of aging. The significant increase in PRA on Day 97 compared to baseline in cats of the RI‐DCN group, coupled with the finding of increased UACR at study end, signals activation of the circulating RAAS after renal injury in this group. Future studies should be conducted on older adult cats to avoid the potentially confounding effect of life stage transition.

Circulating angiotensin II, the main effector peptide of the RAAS, was not significantly different between healthy cats and cats with induced CKD. Studies evaluating the circulating and intrarenal RA(A)S in cats or dogs with kidney diseases have been comprehensively reviewed elsewhere (Huang et al., 2024a). While some studies report activation of the circulating RAAS in cats with CKD (Kai et al., 2022; Mathur et al., 2004; Mishina et al., 1998; Watanabe & Mishina, 2007), others signal a more complex and mixed picture, with RAAS activation depending on the markers being evaluated and the hypertensive status of the examined cats (Jensen et al., 1997; Jepson et al., 2014; Huang et al., 2024b; Steele et al., 2002; Ward et al., 2022). Importantly, circulating concentrations of angiotensin I, II, and III were not significantly correlated to their tissular counterparts in our study, which emphasizes that intrarenal concentrations of angiotensin peptides might not be accurately inferred from their circulating levels. Given the importance of the intrarenal RAS in kidney diseases (Kobori et al., 2007; Yang & Xu, 2017), specific evaluation of this system should be central to research evaluating how the RA(A)S interacts with kidney diseases. However, as direct evaluation of renal tissular peptide concentrations requires invasive techniques, less invasive indicators of intrarenal RAS activity will be crucial for relevant research, particularly studies involving clinical patients. Urinary angiotensinogen has been proposed as a promising candidate and is now increasingly used as a marker of intrarenal RAS activation in studies of people with kidney diseases (Carrara et al., 2014; Cherney et al., 2014; Chou et al., 2018; Jung & Yoo, 2021; Kim et al., 2021; Kurultak et al., 2014; Matsuyama et al., 2018; Tiryaki et al., 2016; Tu & Pratt, 2013).

We were unable to identify significant differences in the renal tissular concentrations of angiotensin peptides between kidneys of the healthy control group and kidneys of the RI or RI‐DCN groups. This conflicts with the findings of a previous study evaluating cats with naturally occurring CKD, in which significantly higher angiotensin I concentrations were found in the kidneys of cats with CKD relative to those of healthy controls (Lourenço et al., 2022). However, in that same study, intrarenal angiotensin II concentrations did not differ between groups (Lourenço et al., 2022). In rodents, some studies involving remnant kidney models have demonstrated increased angiotensin II‐positive cells (Vaziri et al., 2007) or increased angiotensin II concentrations in tissue homogenates following renal injury (Kujal et al., 2010), while another noted lower angiotensin II concentrations in tissue homogenates of rats after 5/6 renal ablation compared to sham‐operated controls (Mackie et al., 2001).

Compared to healthy control tissues, renal mRNA transcription of *ACE*, *AT1R*, and *REN* was lower for both CKD model groups, while transcription of *AGT* was greater in the ischemic kidney of the RI‐DCN group. Lower *ACE* and higher *AGT* transcription match our previous findings in cats with naturally occurring CKD (Lourenço et al., 2022). In human patients with impaired renal function, urinary angiotensinogen concentration–a proposed marker of intrarenal RAS activity, as discussed above–is negatively correlated with renal function (Juretzko et al., 2017; Kobori et al., 2008; Mills et al., 2012). Furthermore, urinary angiotensinogen correlates with both renal inflammation (Dou et al., 2019; Wu et al., 2019) and fibrosis (Ohashi et al., 2017) and predicts future renal function decline (Cui et al., 2018; Dou et al., 2019; Jang et al., 2012; Yamamoto et al., 2007). Although its main source is debated (Kukida et al., 2021; Sun et al., 2020), intrarenal *AGT* expression is a possible source of urinary angiotensinogen (Jang et al., 2012; Nishiyama & Kobori, 2018). Our finding of increased *AGT* transcription in cats with surgically induced CKD suggests that urinary angiotensinogen could be a valid biomarker for kidney diseases in cats, as well.

In both cats and dogs, immunohistochemical studies have demonstrated less abundant ACE signals in animals with impaired kidney function compared to normal controls (Mitani et al., 2014). Decreased ACE staining has also been reported in human beings with nephrosclerosis (Wang et al., 2011); however, it is noteworthy that renal mRNA and protein expression of ACE were not correlated in the same study (Wang et al., 2011). This finding mirrors that of another study using a rat subtotal nephrectomy model (Velkoska et al., 2010). Therefore, it should not be assumed that renal mRNA levels of *ACE* (or any gene, for that matter) directly reflect protein expression levels.

Intrarenal angiotensin I concentration and renal *AGT* and *ACE* transcript levels were independently correlated with serum creatinine concentration in the present study. These correlations were consistent with our inter‐group comparisons, which demonstrated that *ACE* transcription was significantly lower, *AGT* transcription was significantly higher, and intrarenal angiotensin I concentration was numerically higher in one or both CKD model groups. On the other hand, the only parameter that independently correlated with intrarenal angiotensin II was intrarenal angiotensin III, a downstream peptide in the RAS cascade. This emphasizes again that the other parameters evaluated in this study (i.e., circulating RAAS parameters, intrarenal RAS gene transcription, and routine blood work) are unlikely to accurately represent the intrarenal concentration of angiotensin II.

This study has several limitations. First, the study is retrospective in its nature, as we used samples banked from prior studies. Group differences in sex distribution and median age, which are known to affect the RAAS (Javadi et al., 2004), might have been a confounding factor in the present study. Protocols for blood pressure measurement and diet also differ among groups due to the retrospective nature. Blood pressure was determined using different methods in the two CKD groups and, therefore, was not compared across groups. A difference in diet with regard to sodium content between the RI‐DCN group and RI and juvenile control groups is notable, especially given several findings were unique to cats of the RI‐DCN group, and serum electrolyte concentrations differed significantly across groups (although not in a clinically meaningful manner, as all values were within the laboratory's reference interval). The different findings from the RI‐DCN group might also be explained by the more severe renal functional impairment induced by RI‐DCN compared to the RI model; indeed, whereas most cats in the RI group were not, the majority of cats in the RI‐DCN group were azotemic. Differences could also be explained by differences in the timepoint of kidney collection (i.e., 9 months vs. 6 months) post‐renal ischemia, or that the contralateral kidney was not present at study end in the RI‐DCN group. Meanwhile, as the sodium intake was lower in the RI‐DCN group, one would expect this to activate their circulating RAAS (Buranakarl et al., 2004). Finding no difference between the RI‐DCN and control group despite this is, therefore, indicative of a conclusion that renal injuries did not activate circulating RAAS at the timepoints evaluated. The small sample size also makes the statistical analysis prone to type‐2 error. Finally, direct measurement of circulating ACE and ACE2 activity was not conducted in this study due to limited sample volume. Instead, a surrogate for ACE activity, ACE‐S, was calculated.

In conclusion, we demonstrated that circulating concentrations of angiotensin peptides were not significantly correlated with their renal counterparts, and alterations in key intrarenal RAS genes' transcription could not be explained by changes in the circulating RAAS. Compared to healthy cats, cats with surgically induced CKD had lower intrarenal mRNA levels of *ACE*, *AT1R*, and *REN*; and higher intrarenal mRNA levels of *AGT*. Future research in cats and human beings should consider the intrarenal RAS in CKD and the interaction between intrarenal and circulating RA(A)S.

## FUNDING INFORMATION

The authors received no financial support for the research, authorship, and/or publication of this article.

## CONFLICT OF INTEREST STATEMENT

Bianca Lourenço has received research funding from Ceva Animal Health, Boehringer Ingelheim Vetmedica GmbH, Elanco Animal Health, the Translational RAAS Interest Group, which is sponsored by Ceva Animal Health, and speaker honoraria from Boehringer Ingelheim Vetmedica, Ceva Animal Health, Idexx Ltd., Antech Diagnostics, and Nestlé Purina. Chad Schmiedt has received research funding from Ceva Animal Health, Elanco Animal Health, and Boehringer Ingelheim Vetmedica, and a donation from Dechra Veterinary Products. Jamie Tarigo has received research funding from Elanco Animal Health. Amanda Coleman has served as a consultant for and received speaker honoraria from Boehringer Ingelheim Vetmedica GmbH, and received research funding from Boehringer Ingelheim Vetmedica GmbH, Elanco Animal Health, and the Translational RAAS Interest Group. Jane Huang has nothing to declare.

## ETHICS STATEMENT

The study reported here was exempt from institutional animal care and use committee review because only banked blood, urine, and tissue specimens were used; no live animals were involved in the study.

## PERMISSION TO REPRODUCE MATERIAL FROM OTHER SOURCES

The authors used BioRender for generation of Figure 1. A publication license was obtained and is cited in the figure caption.

## Supporting information


Figure S1.

Table S1.

Table S2.

Table S3.

Table S4.

## Data Availability

All data are shared within this manuscript and its Supporting Information.
