# Supplementary material for: Post hoc comparison of the intrarenal and circulating renin‐angiotensin(‐aldosterone) systems in cats with ischemia‐induced chronic kidney disease
Source: Physiol Rep. 2025 Jun 25;13(12):e70417. doi: 10.14814/phy2.70417 (PMC12190553; doi:10.14814/phy2.70417)

**Figure S1.** Comparisons between ischemic and non-ischemic kidneys for cats in the RI group and the RI-DCN group. All RA(A)S components were log-transformed to approximate normal distribution. Pairwise comparisons were conducted using the paired t-test in R (version 4.3.3) package ggplot2 (version 3.5.1) and package ggsignif (version 0.6.4), with the resulting p-values shown above the brackets.
Abbreviations: *ACE*, angiotensin-converting enzyme mRNA; Ang, angiotensin; *AGT*, angiotensinogen mRNA; *AT1R*, angiotensin type-1 receptor mRNA; IK, ischemic kidney; NIK, non-ischemic kidney; *REN*, renin mRNA; RI, unilateral renal ischemia; RI-DCN, unilateral renal ischemia followed by delayed contralateral nephrectomy.


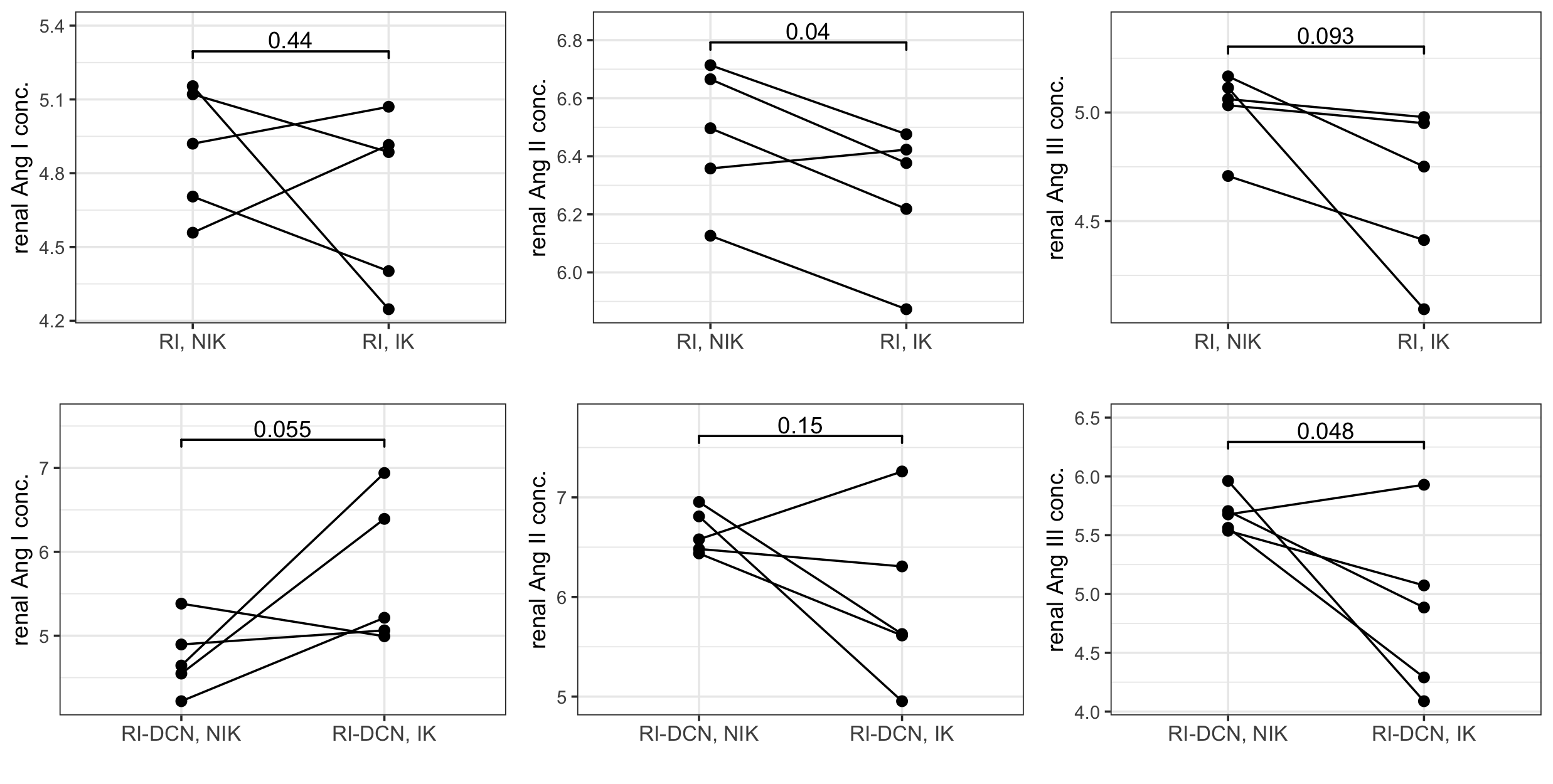


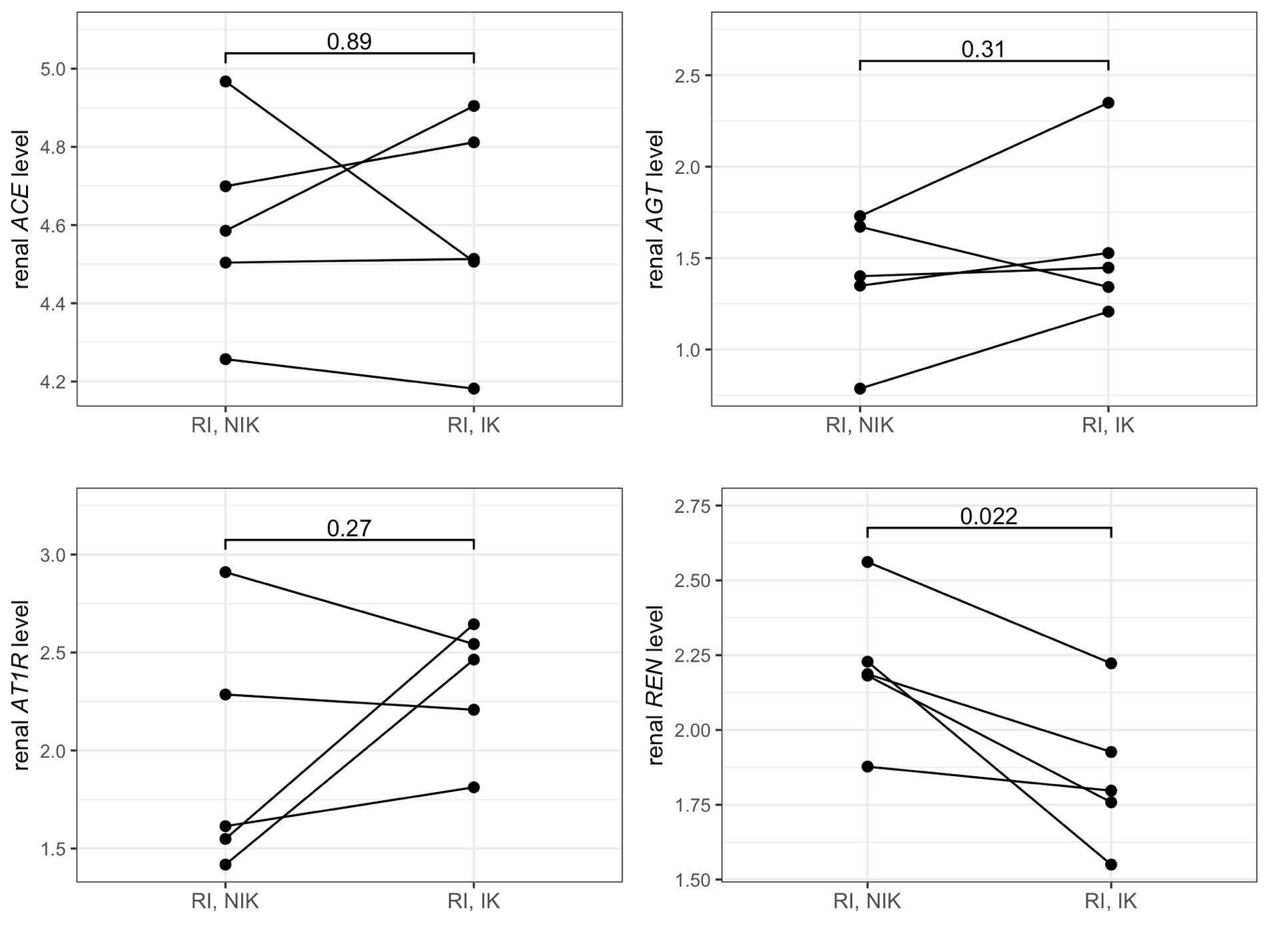


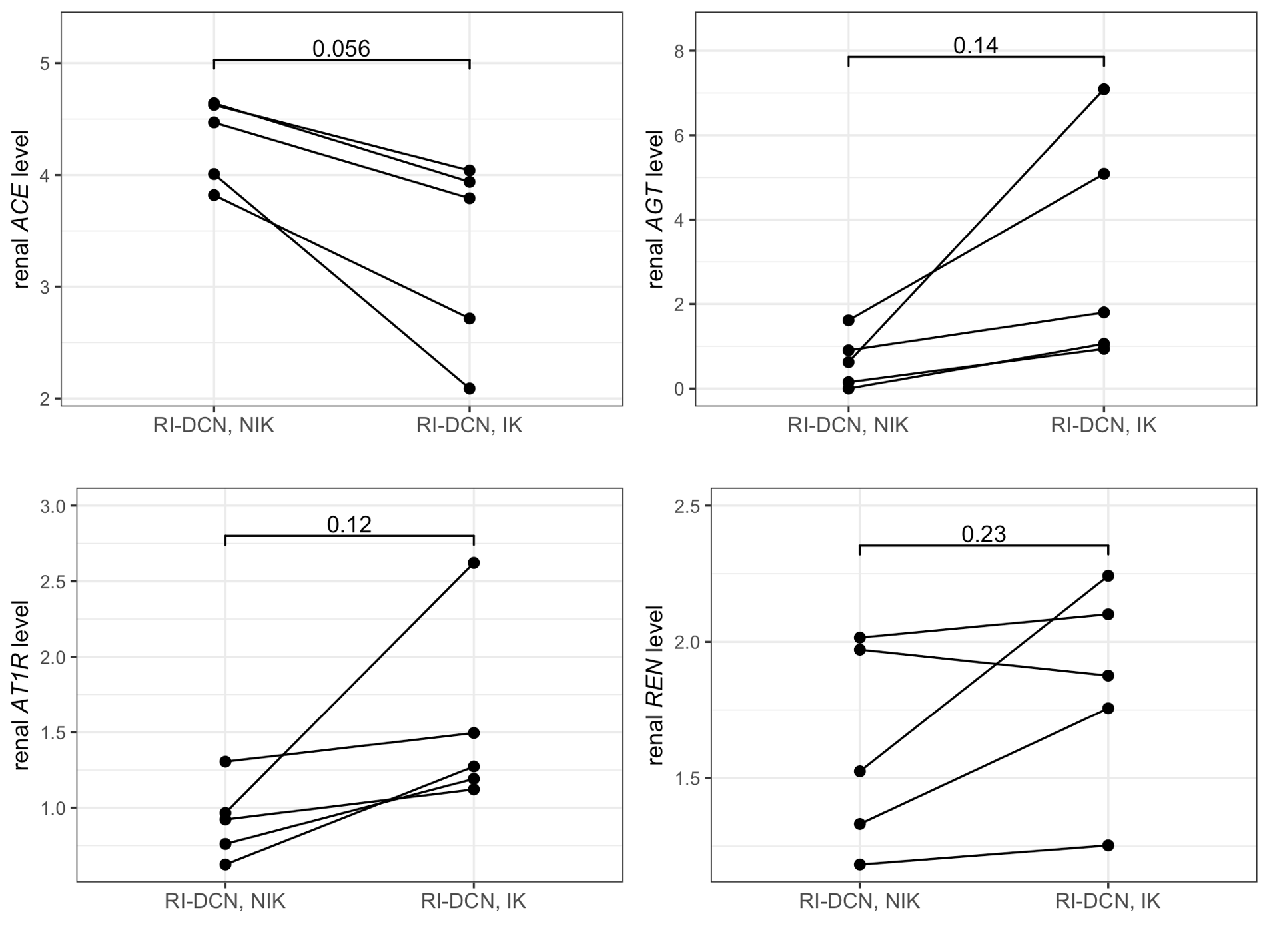

Supplement: Supplementary file 1 — Figure S1. [file PHY2-13-e70417-s003.docx]
