# Supplementary material for: Post hoc comparison of the intrarenal and circulating renin‐angiotensin(‐aldosterone) systems in cats with ischemia‐induced chronic kidney disease
Source: Physiol Rep. 2025 Jun 25;13(12):e70417. doi: 10.14814/phy2.70417 (PMC12190553; doi:10.14814/phy2.70417)
Supplement: Supplementary file 2 — Table S1. [file PHY2-13-e70417-s001.docx]

Table S1 Llinear mixed models for comparing circulating RAAS parameters between control and the two CKD groups. Values of all RAAS components were log-transformed to approximate normal distribution. Groups combined with study timepoints (i.e., control, RI Day 180, RI-DCN Baseline, RI-DCN Day 269) were set as fixed effect; individual cats were set as random effect; and the parameter of interest (i.e., RA[A]S components) were set as the outcome. The models were built with all data points included, using R (version 4.3.3) package lme4 (version 1.1-35.2). Once the models were built, pairwise comparisons between CKD grroups and control group were conducted using package emmeans (version 1.10.1).
Abbreviations: AA2, surrogate measure of adrenal responsiveness to Ang II in serum; ACE-S, surrogate for angiotensin-converting enzyme activity in serum; CKD, chronic kidney disease; df, degree of freedom; REN-S, surrogate for renin activity in serum; RI, unilateral renal ischemia; RI-DCN, unilateral renal ischemia followed by delayed contralateral nephrectomy; SE, Standardized error.

| Serum Ang I | | | | |
| --- | --- | --- | --- | --- |
| contrast | **estimate** | **SE** | **df** | p **value** |
| RI Day180 - Control | 0.327 | 0.517 | 15.693 | 0.837 |
| (RI-DCN Baseline) - Control | -0.111 | 0.578 | 15.693 | 0.985 |
| (RI-DCN Day269) - Control | -0.009 | 0.578 | 15.693 | 1.000 |

| Serum Ang II | | | | |
| --- | --- | --- | --- | --- |
| contrast | **estimate** | **SE** | **df** | p **value** |
| RI Day180 - Control | -0.412 | 0.382 | 16.000 | 0.577 |
| (RI-DCN Baseline) - Control | -0.455 | 0.427 | 16.000 | 0.586 |
| (RI-DCN Day269) - Control | 0.321 | 0.427 | 16.000 | 0.773 |

| Serum Ang III | | | | |
| --- | --- | --- | --- | --- |
| contrast | **estimate** | **SE** | **df** | p **value** |
| RI Day180 - Control | 0.397 | 0.774 | 13.897 | 0.892 |
| (RI-DCN Baseline) - Control | -0.840 | 0.865 | 13.897 | 0.645 |
| (RI-DCN Day269) - Control | 0.455 | 0.865 | 13.897 | 0.886 |

| Serum Ang IV | | | | |
| --- | --- | --- | --- | --- |
| contrast | **estimate** | **SE** | **df** | p **value** |
| RI Day180 - Control | 0.271 | 0.503 | 15.873 | 0.881 |
| (RI-DCN Baseline) - Control | -0.364 | 0.562 | 15.873 | 0.829 |
| (RI-DCN Day269) - Control | 0.205 | 0.562 | 15.873 | 0.946 |

| Serum Ang 1-5 | | | | |
| --- | --- | --- | --- | --- |
| contrast | **estimate** | **SE** | **df** | p **value** |
| RI Day180 - Control | -3.075 | 0.808 | 13.342 | 0.006 |
| (RI-DCN Baseline) - Control | -0.961 | 0.904 | 13.342 | 0.590 |
| (RI-DCN Day269) - Control | 0.875 | 0.904 | 13.342 | 0.648 |

| Serum Ang 1-7 | | | | |
| --- | --- | --- | --- | --- |
| contrast | **estimate** | **SE** | **df** | p **value** |
| RI Day180 - Control | -0.579 | 0.427 | 16.000 | 0.417 |
| (RI-DCN Baseline) - Control | -0.659 | 0.478 | 16.000 | 0.404 |
| (RI-DCN Day269) - Control | 0.054 | 0.478 | 16.000 | 0.995 |

| Serum Aldosterone | | | | |
| --- | --- | --- | --- | --- |
| contrast | **estimate** | **SE** | **df** | p **value** |
| RI Day180 - Control | 0.074 | 0.374 | 16.000 | 0.985 |
| (RI-DCN Baseline) - Control | 0.533 | 0.374 | 16.000 | 0.381 |
| (RI-DCN Day269) - Control | 0.771 | 0.335 | 16.000 | 0.090 |

| ACE-S | | | | |
| --- | --- | --- | --- | --- |
| contrast | **estimate** | **SE** | **df** | p **value** |
| RI Day180 - Control | -0.739 | 0.279 | 13.672 | 0.051 |
| (RI-DCN Baseline) - Control | -0.343 | 0.312 | 13.672 | 0.568 |
| (RI-DCN Day269) - Control | 0.330 | 0.312 | 13.672 | 0.594 |

| REN-S | | | | |
| --- | --- | --- | --- | --- |
| contrast | **estimate** | **SE** | **df** | p **value** |
| RI Day180 - Control | -0.105 | 0.423 | 15.975 | 0.975 |
| (RI-DCN Baseline) - Control | -0.344 | 0.472 | 15.975 | 0.787 |
| (RI-DCN Day269) - Control | 0.249 | 0.472 | 15.975 | 0.886 |

| AA2 | | | | |
| --- | --- | --- | --- | --- |
| contrast | **estimate** | **SE** | **df** | p **value** |
| RI Day180 - Control | 1.184 | 0.449 | 14.220 | 0.051 |
| (RI-DCN Baseline) - Control | 0.528 | 0.502 | 14.220 | 0.596 |
| (RI-DCN Day269) - Control | 0.212 | 0.502 | 14.220 | 0.927 |
