# Supplementary material for: Post hoc comparison of the intrarenal and circulating renin‐angiotensin(‐aldosterone) systems in cats with ischemia‐induced chronic kidney disease
Source: Physiol Rep. 2025 Jun 25;13(12):e70417. doi: 10.14814/phy2.70417 (PMC12190553; doi:10.14814/phy2.70417)
Supplement: Supplementary file 3 — Table S2. [file PHY2-13-e70417-s002.docx]

Table S2 Linear mixed models for comparing intra-renal RAS parameters between the right kidneys of the control group, and the two kidneys in two CKD groups. Kidneys (ischemic or non-ischemic kidney of RI or RI-DCN groups, right kidney of the control group) were set as fixed effect; individual cats were set as random effect; and the parameter of interest (i.e., RAS components) were set as the outcome. Values of RAS components were all log-transformed to improve the approximation of normal distribution. The models were built with all data points included, using R (version 4.3.3) package lme4 (version 1.1-35.2). Once the models were built, pairwise comparisons between ischemic or non-ischemic kidney of RI or RI-DCN groups versus right kidney of the control group were conducted using package emmeans (version 1.10.1).
Abbreviations: ACE, angiotensin-converting enzyme; AGT, angiotensinogen; AT1R, angiotensin type-1 receptor; CKD, chronic kidney disease; df, Degree of freedom; (N)IK, (Non-)Ischemic kidney; RI-DCN, unilateral renal ischemia followed by delayed contralateral nephrectomy; RI, unilateral renal ischemia; RK, Right kidney; SE, Standardized error.

| Renal *ACE* levels | | | | |
| --- | --- | --- | --- | --- |
| contrast | **estimate** | **SE** | **df** | p **value** |
| RI-DCN, IK – Control, RK | -1.959 | 0.264 | 20.002 | <0.001 |
| RI-DCN, NIK – Control, RK | -0.961 | 0.264 | 20.002 | 0.006 |
| RI, IK – Control, RK | -0.868 | 0.250 | 20.002 | 0.009 |
| RI, NIK – Control, RK | -0.774 | 0.261 | 21.353 | 0.025 |

| Renal *AGT* levels | | | | |
| --- | --- | --- | --- | --- |
| contrast | **estimate** | **SE** | **df** | p **value** |
| RI-DCN, IK – Control, RK | 1.822 | 0.682 | 23.384 | 0.046 |
| RI-DCN, NIK – Control, RK | -0.714 | 0.682 | 23.384 | 0.662 |
| RI, IK – Control, RK | 0.314 | 0.646 | 23.384 | 0.936 |
| RI, NIK – Control, RK | 0.037 | 0.689 | 23.691 | 1.000 |

| Renal *AT1R* levels | | | | |
| --- | --- | --- | --- | --- |
| contrast | **estimate** | **SE** | **df** | p **value** |
| RI-DCN, IK – Control, RK | -0.765 | 0.247 | 23.727 | 0.018 |
| RI-DCN, NIK – Control, RK | -1.390 | 0.247 | 23.727 | <0.001 |
| RI, IK – Control, RK | 0.140 | 0.234 | 23.727 | 0.898 |
| RI, NIK – Control, RK | -0.336 | 0.250 | 23.867 | 0.481 |

| Renal *REN* levels | | | | |
| --- | --- | --- | --- | --- |
| contrast | **estimate** | **SE** | **df** | p **value** |
| RI-DCN, IK – Control, RK | -0.594 | 0.166 | 20.091 | 0.007 |
| RI-DCN, NIK – Control, RK | -0.835 | 0.166 | 20.091 | <0.001 |
| RI, IK – Control, RK | -0.590 | 0.157 | 20.091 | 0.004 |
| RI, NIK – Control, RK | -0.233 | 0.164 | 21.433 | 0.435 |

| Renal Ang I conc. | | | | |
| --- | --- | --- | --- | --- |
| contrast | **estimate** | **SE** | **df** | p **value** |
| RI-DCN, IK – Control, RK | 0.699 | 0.294 | 24.000 | 0.085 |
| RI-DCN, NIK – Control, RK | -0.283 | 0.294 | 24.000 | 0.713 |
| RI, IK – Control, RK | -0.318 | 0.279 | 24.000 | 0.604 |
| RI, NIK – Control, RK | -0.130 | 0.298 | 24.000 | 0.950 |

| Renal Ang II conc. | | | | |
| --- | --- | --- | --- | --- |
| contrast | **estimate** | **SE** | **df** | p **value** |
| RI-DCN, IK – Control, RK | -0.479 | 0.253 | 24.000 | 0.211 |
| RI-DCN, NIK – Control, RK | 0.221 | 0.253 | 24.000 | 0.764 |
| RI, IK – Control, RK | -0.127 | 0.239 | 24.000 | 0.921 |
| RI, NIK – Control, RK | 0.041 | 0.256 | 24.000 | 0.995 |

| Renal Ang III conc. | | | | |
| --- | --- | --- | --- | --- |
| contrast | **estimate** | **SE** | **df** | p **value** |
| RI-DCN, IK – Control, RK | -0.318 | 0.253 | 24.000 | 0.534 |
| RI-DCN, NIK – Control, RK | 0.518 | 0.253 | 24.000 | 0.162 |
| RI, IK – Control, RK | -0.394 | 0.240 | 24.000 | 0.319 |
| RI, NIK – Control, RK | -0.154 | 0.257 | 24.000 | 0.895 |
