# Supplementary material for: Post hoc comparison of the intrarenal and circulating renin‐angiotensin(‐aldosterone) systems in cats with ischemia‐induced chronic kidney disease
Source: Physiol Rep. 2025 Jun 25;13(12):e70417. doi: 10.14814/phy2.70417 (PMC12190553; doi:10.14814/phy2.70417)
Supplement: Supplementary file 5 — Table S4. [file PHY2-13-e70417-s005.docx]

Table S4. Multiple linear regression models with serum creatinine (A) or intra-renal angiotensin II (B) as the outcome; log-transferred RA(A)S parameters who correlated with serum creatinine significantly under Pearson correlation analysis as independent variables. Data were used if biological samples from the individual cat were collected within 24 hours of one another, and parameter values across all groups were combined. Statistical analyses were performed with R (version 4.3.3).
*ACE*, angiotensin-convertin enzyme*; AGT*, angiotensinogen; Ang, angiotensin; PRA, Plasma renin activity.

| 1. Serum Creatinine | | | | |
| --- | --- | --- | --- | --- |
|  | Estimate | Std. Error | t value | p-value |
| Serum AngII | -0.281 | 0.423 | -0.665 | 0.521 |
| Serum Ang1-7 | 0.351 | 0.407 | 0.862 | 0.409 |
| Kidney AngI | 0.408 | 0.180 | 2.270 | 0.047* |
| *AGT* | 0.238 | 0.100 | 2.365 | 0.040* |
| *ACE* | -0.421 | 0.162 | -2.597 | 0.027* |

| 1. Intra-renal Angiotensin II | | | | |
| --- | --- | --- | --- | --- |
|  | Estimate | Std. Error | t value | p-value |
| PRA | -0.018 | 0.146 | -0.120 | 0.906 |
| Serum AngI | 0.215 | 0.117 | 1.842 | 0.090 |
| Kidney AngIII | 0.648 | 0.220 | 2.950 | 0.012* |
